# Time-resolved instantaneous functional loci estimation (TRIFLE): Estimating time-varying allocation of spatially overlapping sources in functional magnetic resonance imaging

**DOI:** 10.1162/IMAG.a.58

**Published:** 2025-06-27

**Authors:** Tamara Jedidja de Kloe, Zahra Fazal, Nils Kohn, David Gordon Norris, Ravi Shankar Menon, Alberto Llera, Christian Friedrich Beckmann

**Affiliations:** Donders Institute, Radboud University, Nijmegen, The Netherlands; Department for Cognitive Neuroscience, Radboud University Medical Center Nijmegen, Nijmegen, the Netherlands; Erwin L. Hahn Institute, University Duisburg-Essen, Essen, Germany; Robarts Research Institute, London, Ontario, Canada; Oxford Centre for Integrative Neuroimaging, FMRIB, University of Oxford, Oxford, United Kingdom

**Keywords:** time-varying functional connectivity, dynamic functional connectivity, independent component analysis, temporal functional modes, spatial overlap, functional magnetic resonance imaging

## Abstract

In functional magnetic resonance imaging, multivariate proxies of functional brain networks are commonly extracted using spatial independent component analysis. The theoretical premises of spatial overlap among functional processes and the time-varying nature of functional connectivity prompt the question of how to accurately model spatially overlapping and time-varying functional sources. Well-known functional networks have previously been shown to divide into spatially overlapping and functionally distinct subprocesses termed*Temporal Functional Modes (TFM)*using temporal independent component analysis on the time courses obtained via spatial independent component analysis. In this model, spatial and temporal modes of organisation interact through a single mixing matrix with fixed coefficients. Here, we introduce a time-resolved version termed*Time-Resolved Instantaneous Functional Loci Estimation (TRIFLE)*to estimate time-varying changes in source allocation. We analytically demonstrate that the originally fixed TFM mixing matrix can be expressed as the temporal average of a time-resolved mixing matrix, which in turn can be obtained in closed form and provides instantaneous estimates of brain network reconfigurations involved in distinct temporal functional modes. We apply TRIFLE to a high-temporal resolution functional magnetic resonance imaging dataset. We demonstrate that spatial source allocation aligns with expectations based on the experimental task design and that successful and unsuccessful trials have different allocation profiles. The proposed method sheds light on the temporal evolution of brain network reconfigurations while explicitly accounting for potential neuroanatomical overlap.

## 1 Introduction

Much like different lenses through which a photographer seeks to capture the world, the choice of analytical method critically determines the questions that can be asked and the limits of what can be observed in studying brain function. For example, the commonly used mass-univariate approach to analysing task-based functional magnetic resonance imaging (fMRI) data is predominantly suited for studying functional*segregation*. As it models associations between different conditions of an experimental design with the blood oxygen level-dependent (BOLD) signal at the single voxel level (Poldrack et al., 2011;Smith, 2004), it precludes examination of integration between spatial sources. Since the discovery that spatially distinct and temporally synchronised brain regions can be differentiated in fMRI (Biswal et al., 1995), a complementary*integrationist*approach has gained traction, focused on probing statistical association between brain regions as a proxy for functional connectivity between them (Friston, 2011). Common functional connectivity methods, for example, seed-based correlation analysis, assume that the dependence structure between brain regions is constant throughout an fMRI scan or experimental condition by retrieving time-averaged estimates of statistical association (Biswal et al., 1995;Fox et al., 2005). While working under this assumption, many significant advances have been made in understanding large-scale functional networks and their relation to cognition (e.g.,Laird et al., 2011), behaviour (e.g.,Smith et al., 2015), human development (e.g.,Uddin, 2010), personality (e.g.,Mulders et al., 2018), psychopathology (e.g.,Anand et al., 2005;Greicius et al., 2007;Lynall et al., 2010;Mulders et al., 2015), and neurological conditions (Damoiseaux et al., 2012;Greicius et al., 2004;Lehmann et al., 2015;Lustig et al., 2003). A recent line of research, however, posits that this fails to capture expected changes in functional connectivity and interactions between, or shifts in dominance of, brain regions or networks (Battaglia et al., 2020;Calhoun et al., 2014;Cutts et al., 2023;Di & Biswal, 2020;Hutchison et al., 2013;Iraji et al., 2021;Liégeois et al., 2017;Lurie et al., 2020;Preti et al., 2017;Sporns et al., 2021;Zalesky et al., 2014). It is argued that the characterisation of time-varying functional connectivity (TVFC) metrics is required to account for such variations.

Considerable progress has been made in elucidating the relationship between time-varying fluctuations in functional connectivity and cognition, behaviour, and mental states (for overviews, see:Cohen, 2018;Gonzalez-Castillo & Bandettini, 2018;Hutchison et al., 2013;Iraji et al., 2021;Lurie et al., 2020;Preti et al., 2017). TVFC measures have been shown to specifically relate to task-based cognitive measures such as fluid intelligence (Liégeois et al., 2019;Vidaurre, 2021), working memory (Braun et al., 2015;Eichenbaum et al., 2021;Shine et al., 2016), cognitive control (Hutchison & Morton, 2015), sustained attention (Mooneyham et al., 2017), and transient mental states such as emotion (Dodero et al., 2016).Vidaurre (2021)found support for the hypothesis that TVFC is less driven by brain structure than time-averaged functional connectivity and may thus reflect momentary neuronal communication fluctuating around a stable functional architecture. Alterations in TVFC have also been identified in several psychiatric conditions (e.g., schizophrenia [Damaraju et al., 2014;Fu et al., 2021;Mahmood et al., 2022;Sendi et al., 2021]; anxiety [Y. Chen et al., 2020;Cui et al., 2020;Yao et al., 2017]; and depression [G. Chen et al., 2022;Demirtaş et al., 2016;Figueroa et al., 2019;Sendi et al., 2021;Wu et al., 2019]), in neurological conditions (e.g., Parkinson’s and Alzheimer’s disease [for a review, seeFilippi et al., 2019]), and in developmental neurodivergence (e.g., autism [Fu et al., 2021;Liu et al., 2020;Q. Wang et al., 2021] and ADHD [Sun et al., 2021;X.-H. Wang et al., 2018]). Given the growing interest in probing interindividual differences over group-level patterns (Bijsterbosch et al., 2021), the ability to capture inter-individual variability in TVFC accurately is becoming increasingly important.

The debates over whether time-varying statistical estimates genuinely represent changes in functional connectivity and whether we can reliably estimate TVFC are still ongoing (Gratton et al., 2018;Hutchison et al., 2013;Lurie et al., 2020;Smith et al., 2012;Vidaurre et al., 2021;Zhang et al., 2018). The controversy is partly due to several methodological challenges in estimating TVFC, especially when using the most common sliding-window correlation approach (Allen et al., 2014;Chang & Glover, 2010;Handwerker et al., 2012;Sakoğlu et al., 2010). Here, the entire scan duration is segmented into smaller windows. For fixed window lengths, such approaches act as temporal filters, hindering the characterisation of transient, non-stationary effects. After the window segmentation, functional connectivity metrics are calculated over successive (overlapping or non-overlapping) windows. Interpreting temporal variations in these metrics is not straightforward for several reasons. Instead of changes in functional organisation, time-varying statistical estimates might reflect changes in the mean or variance of the BOLD signal (Hutchison et al., 2013;Smith et al., 2012). Moreover, some noise sources in fMRI are non-stationary and change as a function of time, which can induce changes in estimates (Hutchison et al., 2013). Spurious fluctuations can also arise when analysing components with wavelengths larger than the window length (Hutchison et al., 2013). Given the inverse relationship between periodicity and frequency, short windows only allow probing high-frequency content. In contrast, functional networks are generally assumed to be dominated by low-frequency oscillations (ranging from 0.01 to 0.1 Hz,Biswal et al., 1995). For example, using a window size of 10 s, we are blind to this entire spectrum, as the lowest frequency that can be captured is 0.1 Hz (1/10 s). Even using rule-of-thumb window sizes from 30 to 60 s (Preti et al., 2017), we do not capture a full oscillatory cycle at the lowest end of this spectrum (0.01 Hz), which takes 100 s. Statistical estimates, moreover, depend on window size. Small windows and/or long TRs result in too few measurements to reliably estimate the correlation between two signals (i.e., poor correlation sampling;Smith et al., 2012). This obscures whether observed changes in functional connectivity metrics are due to actual changes in TVFC or result from sampling variability (i.e. statistical noise;Hindriks et al., 2016;Lurie et al., 2020). Large windows, in contrast, potentially smooth out the time-varying properties of interest (Hindriks et al., 2016). The effects of window size cannot be eliminated, but various efforts are undertaken to minimise them (for an overview, seePreti et al., 2017). Alternatives are, among others, time–frequency analyses (Chang & Glover, 2010), point–process analyses (Tagliazucchi et al., 2012), Hidden Markov models (HMM;Vidaurre et al., 2016,^2017^), and autoregressive models (Liégeois et al., 2017;Rogers et al., 2010).

There are several important considerations regarding analytical TVFC methods. First is whether they take temporal ordering into account. Some methods ignore temporal structure altogether, for example, clustering applied to obtain co-activation patterns such that time points are treated interchangeably. In contrast, some explicitly model the temporal structure, such as Hidden Markov models and Autoregressive models. Other TVFC approaches regard the temporal structure at some stages of the analysis, for example, when estimating the phase coherence of BOLD in LEiDA, but ignore it at other stages, for example, by subsequently applyingk-means clustering (Cabral et al., 2017). A second consideration is which time range is considered, varying from instantaneous estimates to larger time windows used in, for example, sliding window approaches. A third distinction is whether the methods are bivariate or multivariate. Bivariate methods probe time-varying changes in functional connectivity between two regions at a time. Depending on the research question, this might be appropriate. However, it could be argued that the assumed multivariate nature of network interactions, by definition, necessitates a multivariate analytical approach. A more practical advantage of multivariate methods is their greater statistical power, which benefits the reliable detection of time-dependent changes in functional connectivity.

Generally, TVFC methods start with an anatomical or functional parcellation. For the latter, spatial Independent Component Analysis (ICA;Biswal, 1999;Beckmann, 2012;Beckmann & Smith, 2004;Beckmann et al., 2005;Calhoun et al., 2001,^2002^,^2009^;Greicius et al., 2004;Hyvärinen et al., 2001;Kiviniemi et al., 2003;Mckeown et al., 1998) is a common choice. This multivariate, model-free source separation technique assumes that the fMRI signal consists of a linear combination or mixture of signals from different spatially independent sources that show similar BOLD activity and which are generally understood as brain networks.Smith et al. (2012)extended this approach by decomposing network time series identified with spatial ICA using temporal ICA to explicitly account for spatial overlap arising from regions participating in multiple networks (Friston, 1998;Fuster, 2009;Xu, Potenza, & Calhoun, 2013;Xu et al., 2016;Xu, Zhang, et al., 2013) and functional subunits smaller than the spatial resolution. In doing so, they found that well-established functional connectivity networks subdivide into different functional subprocesses that overlap spatially. Essentially, this model considers functional network time series retrieved with spatial ICA as a superposition of contributions from various temporally independent sources termed*Temporal Functional Modes*(TFMs), which are unmixed using temporal ICA (seeSection 2.1). This unmixing consists of rotations and stretches of the spatial time series data, algebraically summarised in the so-called*unmixing matrix*. The product of the unmixing matrix with the spatial ICA time series yields the optimal approximation of the TFMs. Consequently, the product of the inverse of this matrix (called the*mixing matrix*) and the TFM time series provides a reconstruction of the spatial ICA time series. Hence, the mixing matrix is a proxy of the extent to which TFMs weigh on or allocate different spatial sources. In other words, it pertains to the spatial description of the approximated sources, whereas the temporal variation is contained in the associated time series. Notably, the mixing matrix’s description of the mapping between the temporal and spatial modes of organisation has fixed coefficients and is thus*time-invariant*.

Whereas in TFM analysisSmith et al. (2012)assumed that nodes remain spatially fixed and contribute to different TFMs in a time-invariant fashion;Calhoun et al. (2014),Iraji et al. (2019), andIraji et al. (2020)advocated for investigating spatial dynamics, that is, expected changes in the size, shape, or location of spatial sources as well as their time-varying contributions to different functional networks. Addressing spatial dynamics faces similar difficulties as those previously described for examining TVFC due to the intrinsically noisy nature of fMRI data in both the spatial and temporal domains. We, therefore, propose that functional connectivity can be well described by fixed spatial and temporal components, which interact via*time-varying mixing*. Based on this assumption, we introduce a*time-resolved*version of the TFM model termed*Time-Resolved Instantaneous Functional Loci Estimation*(TRIFLE). Specifically, we aim to capture the time-varying changes in spatial source allocation associated with various TFMs by temporally unfolding the mixing process between the spatial sources and the TFMs. We demonstrate analytically that the fixed mixing matrix in classical TFM analysis is the temporal average of a time-resolved mixing matrix, which can be obtained in closed form and provides instantaneous estimates of spatial building blocks allocated by various TFMs (seeSection 2.1for the mathematical derivation). Herewith, we introduce a novel multivariate method that captures dynamic network reconfigurations while accounting for spatial overlap. We applied the novel method to a high-temporal resolution fMRI dataset of participants performing a visuomotor association task, providing a behavioural benchmark. The retrieved network reconfigurations aligned with expectations based on the task design, and we found that specific network reconfiguration profiles relate to task success.

## 2 Methods

### 2.1 TRIFLE analysis pipeline

We propose extending classical TFM analysis to obtain instantaneous estimates of spatial source allocation for various temporal functional processes while accounting for spatial overlap. TFM analysis consists of two stages: a spatial ICA to retrieve functionally connected spatial sources and a subsequent temporal ICA to retrieve TFMs (see steps 1 and 2 ofFig. 1for a schematic representation).

**Fig. 1.**
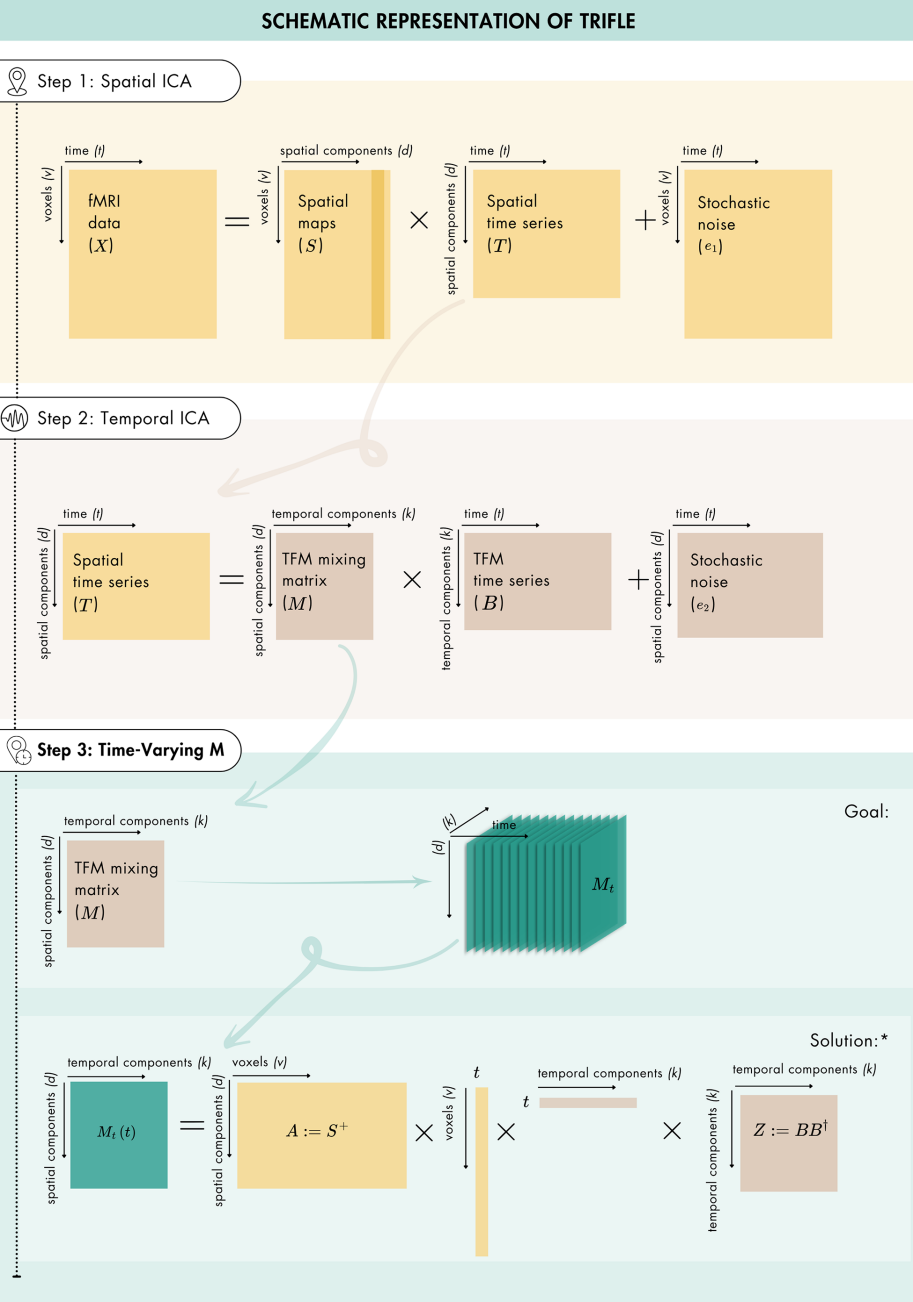
A schematic representation of TRIFLE: that is, steps 1 and 2 comprising TFM analysis, and the novel extension (step 3) where a time-resolved mixing matrix is retrieved. Note that (†) denotes the transpose operation. The spatial pseudoinverse is denoted by ($S^{+}$). (*)This schematic does not include the stochastic noise term of ($M_{t}$) (seeequations 4,5and6).

In step 1, preprocessed fMRI data withvvoxels measured atttime points are decomposed with spatial ICA intodstatistically independent components to identify a set of spatial building blocks. That is, a two-dimensional fMRI data matrix ($X\in {\mathbb{R}}_{v\times t}$) is approximated by a set of*spatially independent*, non-Gaussian sources, which are described as spatial maps ($S\in {\mathbb{R}}_{v\times d}$) and their corresponding time series ($T\;\text{​}\in {\mathbb{R}}_{d\times t}$):

$$X=ST+\epsilon_{1},$$ (1)

where$\epsilon_{1}\in {\mathbb{R}}_{v\times t}$denotes the stochastic noise of the spatial decomposition, which is assumed to be Gaussian distributed and isotropic. All structured noise not removed in the preprocessing stages is part of the model (i.e., ofST).

In step 2, temporal ICA is applied to the spatial ICA time series retrieved in the first step, decomposing them into a set ofkstatistically independent TFMs. That is, the spatial ICA time series are approximated by a set of*temporally independent*non-Gaussian sources, which are described as a product of the TFM mixing matrix ($M\in {\mathbb{R}}_{d\times k}$) and the associated temporally independent time series ($B\in {\mathbb{R}}_{k\times t}$):

$$T\;\text{​}=MB+\epsilon_{2,}$$ (2)

where$\epsilon_{2}\in {\mathbb{R}}_{d\times t}$denotes the stochastic noise of the temporal ICA, which is assumed to be Gaussian distributed and isotropic.

Classical TFM analysis thus describes the preprocessed fMRI data as a product of three matrices: the spatial maps obtained with spatial ICA, the TFM mixing matrix (which describes the extent to which temporal components allocate the spatial components), and the TFM-associated time series:

$$X=SMB+\epsilon_{total},$$ (3)

where$\epsilon_{total}=\epsilon_{1}+S\epsilon_{2}$.

As described inequations (2)and(3), in classical TFM analysis, a*time-invariant*mixing matrix (M) is retrieved. In other words, the extent to which TFMs allocate spatial sources is assumed to be fixed. With TRIFLE, we introduce a third step, extending classical TFM analysis to include time-resolved, instantaneous estimates of the extent to which TFMs allocate spatial sources. That is,*a time-varying*mixing matrix is retrieved (see step 3 ofFig. 1).

Invertingequation (3), the TFM mixing matrixMemerges as a time-averaged quantity:

$$M=AXB^{†}Z+{\tilde{\epsilon }}_{total,}$$ (4)

where†denotes a transpose operation,$A\in {\mathbb{R}}_{d\times v}$refers to the pseudoinverse ofS,$Z\in {\mathbb{R}}_{d\times d}={\left(BB^{†}\right)}^{-1}$so that the product$B^{†}Z$amounts to the right pseudoinverse of the TFM time series. Moreover,${\tilde{\epsilon }}_{total}$concerns a linear projection of$\epsilon_{total}$into the space ofM.

Note that the only time-dependent quantities inequation (4)are$X\in {\mathbb{R}}_{v\times t}$and$B^{†}\in {\mathbb{R}}_{t\times k}$. Considering their product as a sum over time:

M=A(∑t=1TX(t)B†(t))Z+ϵ˜total =∑t=1TAX(t)B†(t)Z+ϵ˜total, (5)

where$X(t)\in {\mathbb{R}}_{v\times 1}$,$B^{†}(t)\in {\mathbb{R}}_{1\times k}$, with:

$$M_{t}(t)=AX(t)B^{†}(t)Z+{\tilde{\epsilon }}_{total}.$$ (6)

In other words, the time-resolved mixing matrix is the element-wise (or Hadamard) product of the participant-specific expression of the network time series (i.e.,AX) and the TFM time series (i.e.,$B^{†}$), scaled throughZ, which represents the covariance ofB. Given the temporal independence of the TFM time series, this covariance is equivalent to the variance ofB. Note that because the structured noise not removed in the preprocessing stages is part ofX, the variance in$M_{t}$will be greater than inM. Additionally, note that for normalised (mean zero and unit variance) time series, the element-wise product amounts to the instantaneous correlation (van Oort et al., 2018). This closed-form solution for$M_{t}$is schematically represented in step 3 ofFigure 1.

The fixed mixing matrix (M), as obtained in classical TFM analysis, can be obtained by summing over the time dimension of this time-dependent quantity:

$$M=\sum_{t=1}^{T}M_{t}(t).$$ (7)

To examine the temporal variation around the fixed mixing matrix values, we define a function:

$$f(t)=NM_{t}(t)$$ (8)

such that

$$M=\frac{1}{N}\sum_{t=1}^{T}f(t).$$ (9)

As shown inequation (9), functionfis a scaled version of$M_{t}$such thatMis the*temporal average*, instead of the sum, of the instantaneous correlations betweenAXand$B^{†}$, scaled by the (co)variance ofB. Herewith, we ensure that we investigate how the allocation of spatial sources*varies around the fixed values of*M.

In summary, step 3 of the TRIFLE model provides us with time-resolved descriptions of the extent to which spatial sources (e.g., canonical functional networks retrieved in step 1) are allocated by various TFMs (retrieved in step 2) using a time-resolved mixing matrix. Remember that the classical TFM model results in fixed spatial descriptions of how each TFM maps onto canonical functional networks and the associated time series, which are proxies of the extent to which different TFMs are involved or “active” over time. We temporally unfold the fixed mapping of each TFM onto the networks to examine the temporal variations in the extent to which spatial sources are allocated for different temporal functional processes. Altogether, we have approximated our data by a set of spatial sources, that is, canonical functional networks (step 1), estimated the overall extent to which these spatial sources are allocated for various TFMs (step 2), and probed how this allocation varies over time (step 3).

A Python package was developed for the TRIFLE method. The package is available athttps://github.com/tamarajedidja/trifle.

### 2.2 Dataset

We used high temporal resolution fMRI data of participants performing three runs of a visuomotor association task in one session. Data were collected at the Robarts Research Institute at the University of Western Ontario in Canada. Healthy volunteers were recruited according to the Health Sciences Research Ethics Board of Western University guidelines. All volunteers provided written informed consent. Data were structured according to the Brain Imaging Data Structure (BIDS;K. J. Gorgolewski et al., 2016).

#### 2.2.1 Sample

Seventeen healthy volunteers participated in this study. Inclusion criteria were right-handedness and age under 50 years. Visual inspection of data quality resulted in the exclusion of three participants due to an acquisition error, that is, the scanner timing was not well matched to the presentation of stimuli. This exclusion led to a final sample of 14 participants between 19 and 32 years, with seven identifying as female (average age of 23.43,*SD*= 3.36) and 7 as male (average age of 24.57 years,*SD*= 4.28). One individual task run was excluded from the analysis for two participants based on insufficient brain coverage.

#### 2.2.2 Procedure

Participants underwent one scanning session lasting approximately 40 min. During this session, they engaged in three runs of a visuomotor association task while fMRI data were acquired, followed by an anatomical scan at the end. Before the scanning session, participants received training on the task by completing one run. As depicted inFigure 2, the task involved learning arbitrary associations between eight Japanese Kanji characters and four different motor responses through trial and error. In each task run, between 25 and 36 visual stimuli (average number of 31.14,*SD*= 1.68) were presented for 200 ms. Participants were instructed to respond as soon as possible but at least within 1.5 s after stimulus onset. Motor responses consisted of pressing one of four buttons on a fibre optic response pad, where each button corresponded to two different Kanji characters. The response pad was placed on the participant’s abdomen, who consistently kept the right hand’s index, middle, ring, and little fingers on corresponding buttons.

**Fig. 2.**
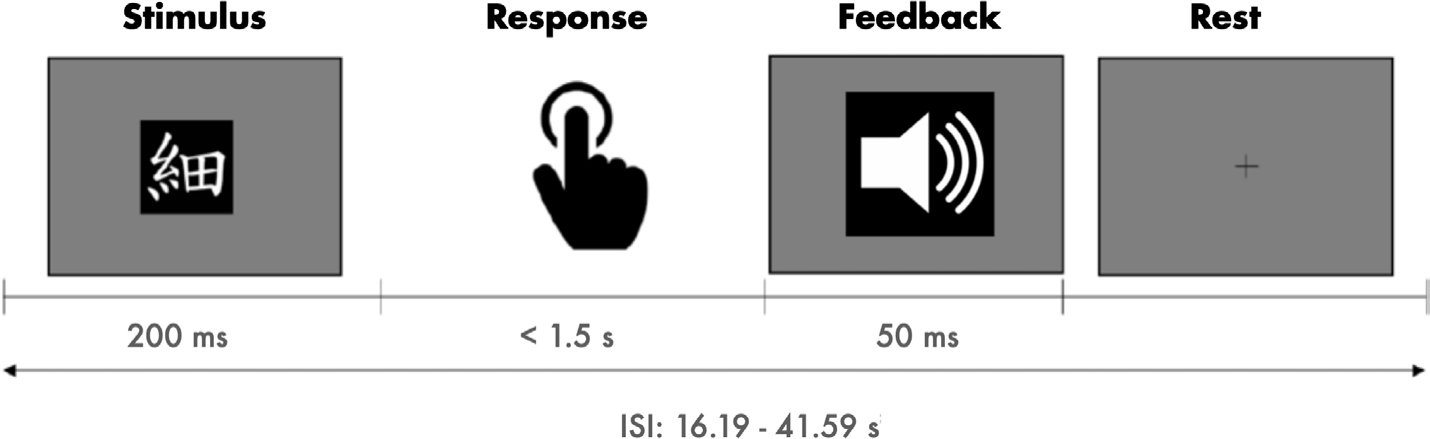
Schematic overview of the visuomotor association task. Visual stimuli were presented for 200 ms, motor responses were required within 1.5 s, immediate auditory feedback was provided for 50 ms, and was followed by a rest period.

Following each response, participants received immediate performance feedback through three distinct sounds indicating a correct, incorrect, or delayed response. Feedback was presented for 50 ms, followed by a fixation cross that remained until the next trial. To ensure a return to baseline of the haemodynamic response before the next trial, long inter-stimulus intervals (ISI) ranging from 16.19 to 41.59 s (average ISI of 19.46,*SD*= 3.28, and 1 outlier of 80.47 s) were used. The presentation of visual stimuli and registration of motor responses were carried out using Presentation 20.1 (Neurobehavioral Systems, San Francisco, CA).

#### 2.2.3 Data acquisition

Data were acquired on a Siemens MAGNETOM 7T MR scanner equipped with a 32-channel head coil. A highly optimised AC84 (GenII) head gradient engine with a maximum gradient strength of 80 mT/m allowed the acquisition of T2* images at an ultrahigh temporal resolution. Functional images were acquired using a 2D multiband gradient-echo EPI protocol with an interleaved slice acquisition sequence (TR = 0.206 s, TE = 22 ms, voxel sizes = 2.70 × 2.70 × 3.11 mm, flip angle = 20°, matrix size = 86 × 86, 32 slices, slice thickness/gap = 2.7 mm/3.1 mm, acceleration factor = 8; seeSupplementary Fig. S1for a single slice image obtained at this temporal resolution). A total of 3000 volumes were obtained per task run of about 10 min. Image reconstruction was performed using Slice-GRAPPA (Cauley et al., 2014;Hoge et al., 2018;Setsompop et al., 2012). For registration purposes, anatomical images were acquired using the MP2RAGE sequence (Marques et al., 2010; TR = 6 s, TE = 2.3 ms, voxel sizes = 1 mm isotropic, flip angles = 4 and 5 degrees; TI1 = 0.8 s, TI2 = 2.7 s; matrix = 240 × 240; 240 slices of 1 mm slice thickness).

#### 2.2.4 Data preprocessing

Data were preprocessed using fMRIPREP version 1.1.6 (Esteban et al., 2019), which is a Nipype-based tool (version 1.1.2;K. Gorgolewski et al., 2011,^2018^).

Anatomical scans were corrected for spatial intensity variations using N4BiasFieldCorrection (ANTs 2.2.0;Tustison et al., 2010). A T1-weighted reference map was computed after registering the T1-weighted images using the mri_robust_template (Freesurfer, 6.0.0;Reuter et al., 2010). The T1-weighted reference was brain extracted using antsBrainExtraction.sh (ANTs 2.2.0) with a target template from the Open Access Series of Imaging Studies. Nonlinear registration to the ICBM152 Nonlinear Asymmetrical template version 2009c (Fonov et al., 2009) was performed using antsRegistration (ANTs 2.2.0;Avants et al., 2008). The preprocessed T1-weighted reference map was used as a reference for functional registration.

The following preprocessing was applied for all task runs. Motion correction parameters were calculated using MCFLIRT (FSL 6.0.0,Jenkinson et al., 2002). A reference volume and its brain-extracted version were generated using a custom methodology of fMRIPREP, that is averaging non-steady-state volumes and computing a brain mask with init_enhance_and_skullstrip_bold_wf. Susceptibility distortions were estimated using a field mapless approach as implemented in ANTs 2.2.0., that is, registering the functional reference image to the T1-weighted reference using intensity inversion (Huntenburg, 2014;S. Wang et al., 2017) while regularising by constraining deformation to be nonzero only along the phase-encoding direction and modulation with an average field map template (Treiber et al., 2016). The transformation parameters for co-registration to the T1-weighted reference were calculated with MCFLIRT (FSL 6.0.0,Jenkinson et al., 2002), using boundary-based registration (Greve & Fischl, 2009) with nine degrees of freedom. A concatenated transform to correct for head motion, susceptibility distortion, functional to T1-weighted registration, and registration from T1-weighted to MNI space (i.e., MNI152NLin2009cAsym) was applied in one step using Lanczos interpolation (Lanczos, 1964), as implemented in antsApplyTransforms. Datasets were temporally high-pass filtered with a cut-off frequency of 0.01 Hz (i.e., a periodicity of 100 s) and spatially smoothed with an FWHM of 5.4 mm. ICA-based denoising of the data was performed. For each task run, the data were decomposed into spatially independent components using Probabilistic ICA (Beckmann & Smith, 2004) as implemented in MELODIC (version 3.15, FSL). The following preprocessing was applied to the input data: masking of non-brain voxels, voxel-wise de-meaning of the data, normalisation of the voxel-wise variance, and whitening and projection into a 70-dimensional subspace using Principal Component Analysis. The whitened observations were decomposed using spatial ICA. Estimated component maps were divided by the standard deviation of the residual noise and thresholded by fitting a mixture model to the histogram of intensity values (Beckmann & Smith, 2004). IC components were manually classified into signal and noise through visual inspection in FSLeyes (McCarthy, 2022), and the preprocessed data were de-noised using fsl_regfilt (FSL v.6.0.0, Jenkinson et al., 2012. Moreover, the preprocessed data were variance normalised (mean zero and unit standard deviation across time) using fslmaths (FSL v.6.0.0,Jenkinson et al., 2012). FollowingGomez et al. (2020), several confounding time series were computed to relate to the TFM time series as an indication of the latter’s neuronal basis (seeSupplementary Fig. S2). This concerned three region-wise global signals extracted within the CSF, the WM, the whole-brain mask, and the head-motion estimates from FSL’s MCFLIRT (Jenkinson et al., 2002).

#### 2.2.5 Trifle

To probe the allocation of known spatial sources, for step 1, we performed dual regression (Beckmann et al., 2009;Nickerson et al., 2017) with respect to previously established functional templates instead of running group spatial ICA (Smith et al., 2012). We used a parcellation of model-order 20 to identify major canonical functional networks (i.e.,*SMITH20*template) and a model-order 70 to identify canonical functional subnetworks (i.e.,*SMITH70*template;Smith et al., 2009). These (sub)networks were previously identified in resting state using group spatial ICA and were shown to correspond to networks identified from a large set of task-based brain activation studies (Smith et al., 2009).

For step 2, we ran temporal ICA on the canonical functional (sub)network time series using sklearn.decomposition.FastICA (Hyvärinen & Oja, 2000) from the scikit-learn 0.24.1 package (Pedregosa et al., 2011) in Python 3.7.10, using a hyperbolic tangent (i.e., “logcosh”) as the nonlinearity for estimating negentropy. FollowingGomez et al. (2020), we ran temporal ICA at the individual level rather than at the group level (Smith et al., 2012) because the ultra-fast fMRI sequence (TR = 0.206 s) provided a sufficient number of samples for stable estimation of TFMs per participant and because it allows us to capture individual-specific dynamics that may be obscured in group-level analyses (i.e., interindividual variation might end up in the residual error term). In the reported analysis, we used a model order of 15 for the network-level parcellation and 21 for the subnetwork-level parcellation. The latter is in line withGomez et al. (2020)andSmith et al. (2012). This second step resulted in time-*invariant*spatial descriptions (e.g., seeSupplementary Figs. S3 and S4) of 15 and 21 TFMs for the SMITH20 and SMITH70 templates, respectively, and the corresponding time series.

As mentioned previously, because structured noise that was not removed in the preprocessing stages is part ofX, the variance in$M_{t}$is larger than inM. Therefore, we computed$M_{t}$/fbased on a denoised reconstruction ofX(i.e., the product ofSMB) in step 3. TRIFLE resulted in time-resolved mixing matrices per task run and spatial network template. We explored task-related variability as represented in the time-resolved mixing matrix for the TFM most strongly associated with the task (i.e.,*task-relevant TFM*). Time-resolved spatial descriptions of the loci allocated by the TFM were retrieved by taking the product of the spatial maps (S) with the time-resolved mixing matrix entries related to that TFM per time point.

The time-resolved mixing matrices were demeaned to probe the temporal properties of (sub)network allocation fluctuations around the fixed magnitudes. Additionally, the direction of the time series is not informative, that is, contingent upon the sign of the time-invariantM. Therefore, we ensured correspondence across task runs and participants by reversing the direction of the time-resolved mixing matrix time series if the temporal correlation of the task-relevant TFM with the task regressors was negative.

#### 2.2.6 Validation

We validated TRIFLE using two approaches. First, we examined whether the temporal order and relative direction of network allocation, as represented in the time-resolved mixing matrix weights, aligned with the task design across participants. Second, we investigated potential differences between successful and unsuccessful trials in canonical functional (sub)network reconfigurations associated with the task-relevant TFMs. It is important to note that the focus here is on the temporal profiles of network allocation rather than their relation to behavioural outcomes.

##### 2.2.6.1 Validation one: task-related network allocation

Statistical testing for the first validation was done in three steps: (1) rank-ordering network weights per time point; (2) computing group-levelZ-values; and (3) parametrisation of the temporal features of network allocation using general linear models (GLM). We segmented the time-resolved mixing matrices into trials for the first two steps to probe the task-evoked spatial source reconfigurations. A trial consisted of a 60-frame epoch (i.e.60×0.206s=12.36s), starting at the onset of the visual stimulus. We chose this interval to cover the expected time it takes for the haemodynamic response to increase in response to the visual stimulus, for planning a motor response, and for the haemodynamic response to return to baseline while not overlapping between trials.

In step 1, as an initial temporal depiction of spatial source reconfigurations, we examined the most strongly recruited canonical functional network per time frame by means of numerically rank ordering the time-resolved mixing matrix weights.

In step 2, we probed whether the time-resolved mixing matrix captures task-relevant information at the group level by examining whether we observed an increasing positive difference from baseline for the visual, sensorimotor, and auditory network weights, respectively, for the task-relevant TFM. Since the DMN is widely recognised as a task-negative network (Gusnard & Raichle, 2001;Raichle et al., 2001;Singh & Fawcett, 2008), we additionally examined whether we observed a relative negative trend from baseline for DMN network weights. Given the exploratory nature of this study, we aimed to represent this in a manner as close as possible to the raw data. Therefore, we conducted independent samplet-tests of the weights against zero per epoch time point across all trials per participant. Group-level standardised (i.e.,Z-) values were subsequently computed.

In step 3, we parametrised the temporal properties of network allocation as represented in the time-resolved mixing matrix within a general linear model framework using FMRIB’s Linear Optimal Basis Set (FLOBS;Woolrich et al., 2004). Using generated basis functions to predict the time series offenables assessing whether the allocation of different networks is relatively shifted in time (mainly specified by the second basis function) and/or more or less dispersed (mainly specified by the third basis function). FLOBS generates many potential HRFs based on random selections of parameters within preset ranges. As temporal properties of network allocation as represented by the time-resolved mixing matrices have not previously been explored, these preset ranges were based on the trial-based group-levelZ-statistics described in the previous paragraph (i.e., onset latency: 0–2.5 s, time from onset to peak: 2.5–6 s, time from peak to undershoot: 2.5–8 s, time from undershoot to baseline: 2.5–6 s). FLOBS subsequently uses principal component analysis to generate an optimal basis set of the generated samples. Time-resolved mixing matrix weights were predicted from the visual task regressor convolved with six basis functions generated with FLOBS per network. We accounted for temporal autocorrelation using a generalised least squares AR(5) model implemented in the statsmodels.linear_model.GLSAR package (McKinney et al., 2011) in Python 3.7.10. The criterion for selecting the autoregressive lag is detailed in the Supplementary Material (seeSupplementary Fig. S5). Multiple comparison correction was applied using the Benjamini–Hochberg false discovery rate (FDR) correction implemented in the statsmodels.stats.multitest.fdrcorrection package with an error rate ofα= 0.05. Linear combinations of the products of the fitted values with the six basis functions provided the temporal profiles of network allocation.

##### 2.2.6.2 Validation two: distinct network allocation profile for task success

Following the same reasoning described before (i.e., aiming to represent the time-resolved functional loci reconfigurations as close as possible to the raw data), statistical testing for the second validation consisted of computing independent samplet-tests between successful and unsuccessful trials per network, per task run, and subsequent calculation of group-level standardised (i.e.,Z–) values.

Differences in spatial source allocation between successful and unsuccessful trials were parametrised within a general linear model framework using FLOBS. That is, time-resolved mixing matrix time series corresponding to the task-relevant TFM were predicted from the six basis functions, a main effect of task success and interactions effects with the visual task regressor convolved with the FLOBS basis functions. Again, an AR(5) model was implemented to account for temporal autocorrelation, and correction for multiple comparisons was implemented with FDR. Linear combinations of the products of fitted values with the six basis functions separately for successful and unsuccessful trials provided the temporal profiles of network allocation.

## 3 Results

### 3.1 Validation one: task-related network allocation

Spatial depictions of the functional loci reconfigurations of the task-relevant TFM retrieved with TRIFLE are shown for a single trial in panel A ofFigure 3(SMITH20 template). Time locked to the visual stimulus, we observed the allocation of primary visual areas, followed by medial and lateral visual areas. Time locked to the motor response, we observed superior parietal motor regions and pre-central gyrus/primary motor cortex allocation. These single-trial data are drawn from what will be referred to as “the selected dataset”. The criteria for selecting these data for illustrative purposes are detailed in theSupplementary Material. A temporal depiction of the functional loci allocations for the same trial is presented in panel B ofFigure 3. It shows the network most strongly allocated by the task-relevant TFM per time point, determined by numerical rank ordering of the time-resolved mixing matrix weights. The ranks closely aligned with the task design: The frontoparietal attention and default-mode networks predominated before the stimulus’s onset. Following the onset of the visual stimulus, the visual networks took precedence, followed by short periods in which the sensorimotor and auditory networks subsequently ranked highest. Averaging the network weights across trials smooths the pattern and eliminates the predominance of the sensorimotor network (see panel C ofFig. 3). A similar pattern was observed at the group level, that is, the most common highest-ranking network per time point across task runs and participants (see panel D ofFig. 3). Here, a predominance of the DMN occurred before the onset of the trial.

**Fig. 3.**
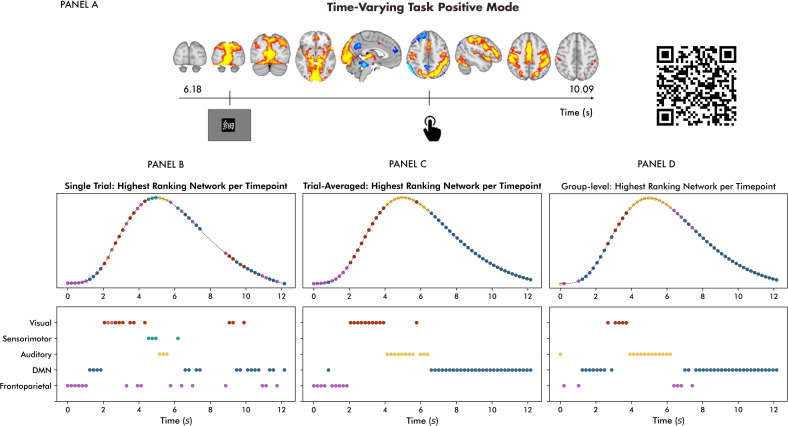
Spatiotemporal depictions of functional loci allocations: Panel (A) shows spatial representations of the time-varying task-relevant TFM for the selected dataset. The onsets of the visual stimulus and motor response are defined as the first time frame where the standardised HRF-convolved stimulus/response exceeds zero. Colour coding: blue (negative) to red to yellow (positive). The QR code links to a video of this trial. Panel (B) shows the highest ranking network per time point. Panel (C) shows the ranking of trial-averaged network weights, and Panel (D) shows group-level results, that is, the most common highest ranking networks across sessions. DMN = default mode network.

Trial-averaged time-resolved mixing matrix weights, illustrating average network allocation across trials, are presented for the selected dataset in panel A ofFigure 4. Refer to the Supplementary Material (seeSupplementary Fig. S6) for an illustration of how the trial-averaged network and TFM time series relate to TRIFLE’s time-resolved mixing of the two. A subsequent allocation of primary visual, sensorimotor, and auditory networks and a relative negative trend from the baseline of the DMN network was observed. In agreement with the eliminated sensorimotor network predominance in the ranking approach (seeFig. 3), the magnitudes of the sensorimotor network weights are relatively small. This is unsurprising, given that the temporal jitter due to varying reaction times is unaccounted for. Therefore, the following group-level analysis was additionally run using trials starting from10×TRbefore the motor response (seeSupplementary Fig. S7). Unsurprisingly, the sensorimotor response was more pronounced here. Apart from that, the results did not substantially differ from those presented below.

**Fig. 4.**
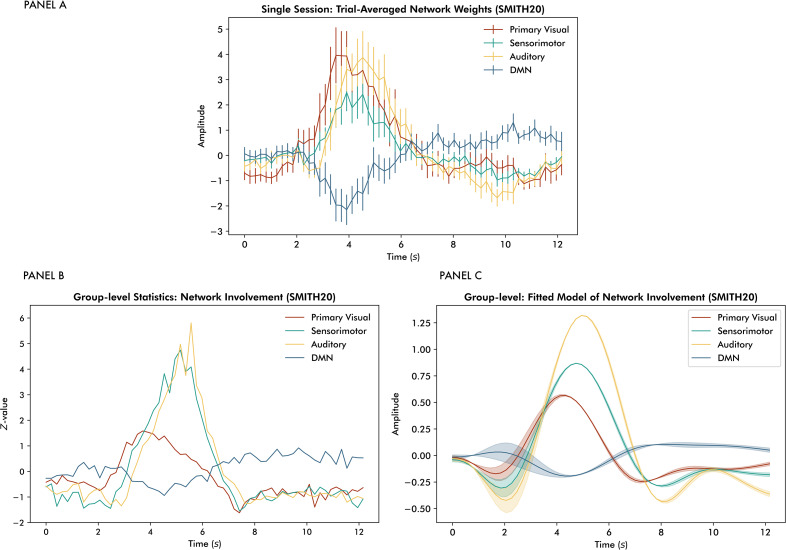
Panel (A) shows trial-averaged time-resolved mixing matrix weights for the task-relevant TFM, depicted for the selected dataset. Error bars are presented per TR of 0.206 s and represent the standard error of the mean. Panel (B) shows group-level differences from baseline for the primary visual, sensorimotor, auditory, and default mode networks, indicated by the red, green, yellow, and blueZ-value time series, respectively. Panel (C) depicts a reconstruction of network allocation based on the general linear model statistics (seeSupplementary Table S1).

At the group level, the temporal order of functional loci allocations corresponding to the task-relevant TFM adhered to the expectations based on the task design (see panel B ofFig. 4). Visual network allocation preceded sensorimotor and auditory network allocation (seeSupplementary Figs. S8 and S9for plots per task run and participant). A reconstruction of the network allocations based on the fittedβ-parameters from the general linear models using FLOBS’ basis functions is presented in panel C ofFigure 4(seeSupplementary Table S1for the model statistics). Correspondence to the group-level statistics in panel B indicates the potential of using this parameterisation of network allocation.

The temporal pattern of network allocation replicates at the subnetwork level, as presented in panel A ofFigure 5. In addition to primary visual network allocation preceding increases in sensorimotor and auditory network weights, a relative deactivation of the precuneus (a core hub of the DMN) in response to the task was observed. The sensorimotor network splits into lateralised parts at the subnetwork level, that is, left and right hemispheres. TRIFLE was found to be sensitive to lateralisation, illustrated by the reconstructions of subnetwork allocation based on the fittedβ-parameters from the general linear models using FLOBS’ basis functions for the left-hemisphere and right-hemisphere sensorimotor networks separately (see panel B ofFig. 5). These findings are consistent with all participants being right-handed, which typically corresponds to a predominant allocation of the sensorimotor network in the left hemisphere. Refer toSupplementary Table S2for an overview of the model statistics.

**Fig. 5.**
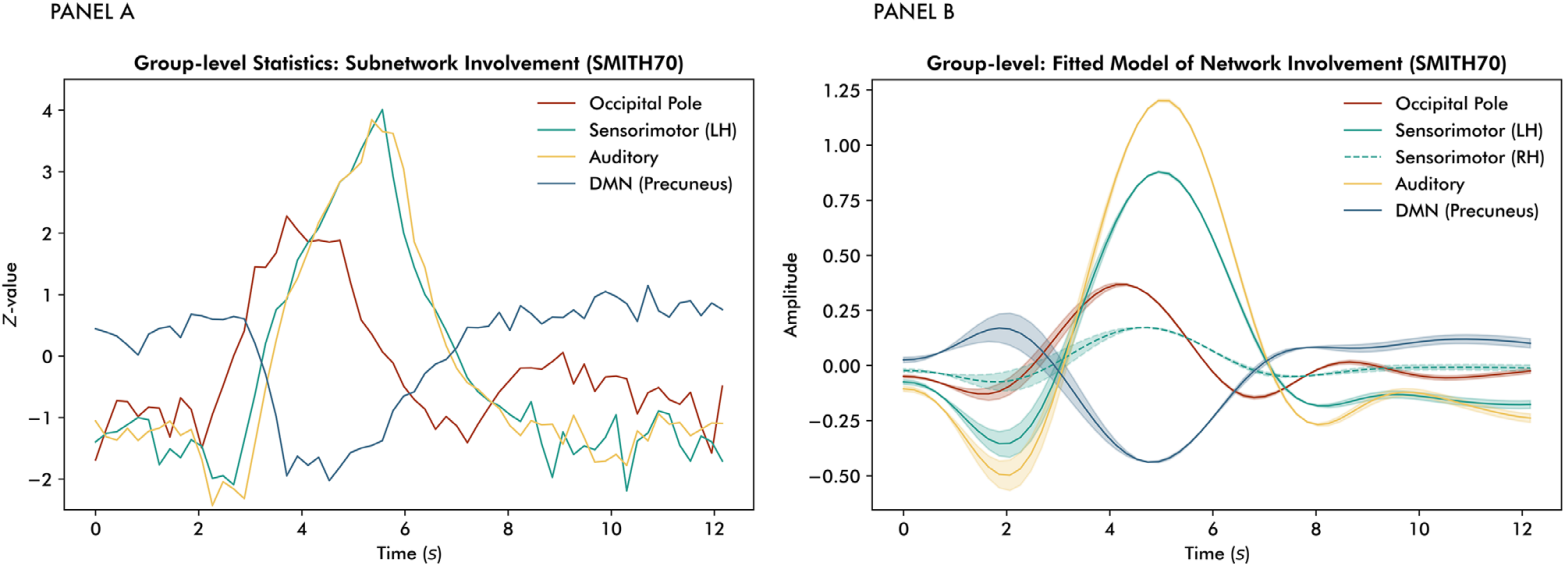
Trial-averaged subnetwork allocation: Panel (A) shows group-level differences from baseline for an occipital, left-hemisphere sensorimotor, auditory and precuneus network, indicated by the red, green, yellow, and blue Z-time series, respectively. Panel (B) depicts a reconstruction of network allocation based on the general linear model statistics (seeSupplementary Table S2).

### 3.2 Validation two: distinct network allocation for task success

In addition to validating whether TRIFLE adequately captured within-trial functional loci allocations consistent with the task design, we explored the network allocation profiles associated with task success. The largest difference in network allocation between successful and unsuccessful trials was observed for the DMN. Panel A ofFigure 6presents the group-level results for the fail–hitt-tests per time point, revealing a stronger suppression of the DMN during unsuccessful trials. This observation was supported by the general linear model (F= 6.74, p<0.001), which yielded a significant post hoc fail–hit contrast and significant post hoc interaction effects between task success (fail–hit) and the first, third, and sixth basis functions (seeTable 1). Panel B ofFigure 6illustrates the corresponding differences in network allocation between successful and unsuccessful trials. As expected, pre-trial variability is considerable. However, a distinct pattern emerges post-stimulus, where the DMN is more strongly suppressed during unsuccessful trials.

**Fig. 6.**
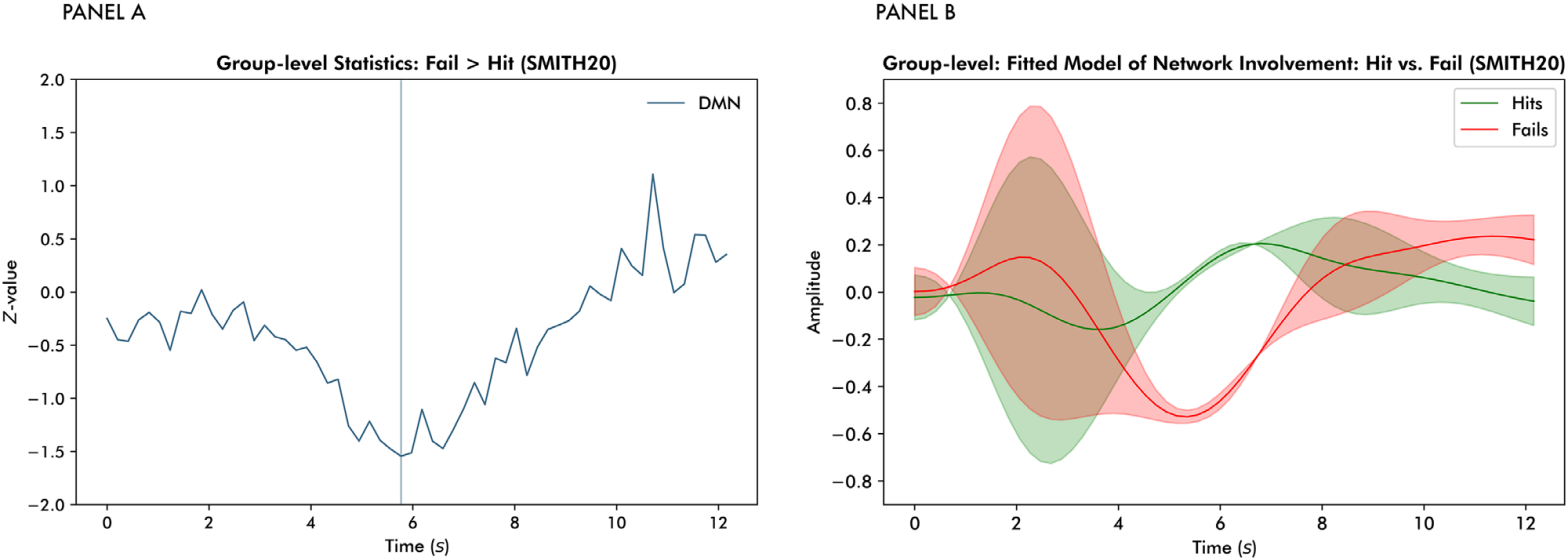
Panel (A) shows the group-level results of the fail–hitt-tests per time point. The vertical line indicates the time point at which the largest difference occurred. Panel (B) presents a reconstruction of differences in default mode network allocation between successful and unsuccessful trials based on the general linear models predicting network allocation from the FLOBS basis functions, task success, and their interactions.

**Table 1.** GLM statistics from models predicting network allocation from FLOBS’ basis functions and task success.

	GLM statistics					Post hoc contrasts fail–hit		
Outcome	Predictor	*b*	*SE*	*t*	*p*	Δb	*t*	*p*
DMN	Hit	0.04	0.030	1.40	0.161			
	Fail	-0.09	0.038	-2.27	0.023	-0.13	-3.03	0.002 *
	BF1	0.03	0.044	0.79	0.433			
	BF1 × Hit	0.00	0.044	-0.04	0.969			
	BF1 × Fail	-0.16	0.050	-3.20	0.001 *	-0.16	-3.77	< 0.001 *
	BF2	0.09	0.072	1.27	0.205			
	BF2 × Hit	-0.17	0.074	-2.29	0.022			
	BF2 × Fail	-0.13	0.077	-1.73	0.084	0.04	1.14	0.256
	BF3	-0.02	0.046	-0.35	0.728			
	BF3 × Hit	0.01	0.048	0.21	0.831			
	BF3 × Fail	0.13	0.051	2.56	0.010 *	0.12	3.89	< 0.001 *
	BF4	-0.05	0.036	-1.39	0.165			
	BF4 × Hit	0.06	0.037	1.55	0.120			
	BF4 × Fail	0.06	0.039	1.59	0.111	0.01	0.23	0.820
	BF5	0.01	0.028	0.44	0.658			
	BF5 × Hit	0.00	0.029	-0.02	0.981			
	BF5 × Fail	0.04	0.032	1.37	0.172	0.04	2.00	0.045
	BF6	0.01	0.029	0.23	0.820			
	BF6 × Hit	0.02	0.030	0.60	0.548			
	BF6 × Fail	-0.04	0.032	-1.16	0.248	-0.06	-2.70	0.007 *

* Significance based on FDR correction (<0.05), originalp-values are presented.

At the subnetwork level, the precuneus, a core hub within the DMN, exhibited the fourth-largest peak difference across networks. Among these four networks, the precuneus reaches its peak difference first, as indicated by the vertical bars in panel A ofFigure 7. Panel A ofFigure 7illustrates the group-level results of the fail–hitt-tests per time point for these four networks. We observed a stronger suppression of the precuneus during unsuccessful trials. This finding was supported by the general linear model predicting precuneus allocation from the FLOBS basis functions, a main effect of task success and their interactions (seeTable 2). Specifically, it yielded a significant post hoc fail–hit contrast and significant post hoc interaction effects between task success (fail–hit) and the first, third, and sixth basis functions. The corresponding differences in the temporal characteristics of network allocation are presented in panel B ofFigure 7. As with the large-scale network level, pre-trial variability is considerable. However, a clear distinction emerges post-stimulus, where the precuneus is more strongly suppressed for unsuccessful trials.

**Fig. 7.**
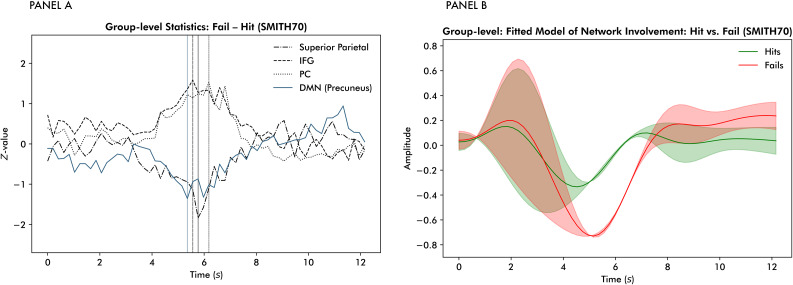
Panel (A) displays the group-level results of the fail–hitt-tests per time point. The vertical lines indicate the time points at which the largest differences occurred. Panel (B) depicts a reconstruction of differences in precuneus allocation between successful and unsuccessful trials based on the general linear model predicting network allocation from the FLOBS basis functions, task success, and their interactions.

**Table 2.** GLM statistics from models predicting subnetwork allocation from FLOBS’ basis functions and task success.

	GLM statistics					Post hoc contrasts fail–hit		
Outcome	Predictor	*b*	*SE*	*t*	*p*	Δ *b*	*t*	*p*
Precuneus (DMN)	Hit	0.05	0.021	2.55	0.011 *			
	Fail	-0.02	0.028	-0.86	0.387	-0.08	-2.45	0.014 *
	BF1	-0.19	0.040	-4.57	< 0.001 *			
	BF1 × Hit	0.11	0.041	2.75	0.006 *			
	BF1 × Fail	-0.03	0.045	-0.59	0.559	-0.14	-4.50	< 0.001 *
	BF2	0.10	0.054	1.88	0.061			
	BF2 × Hit	-0.14	0.055	-2.55	0.011 *			
	BF2 × Fail	-0.13	0.057	-2.24	0.025	0.01	0.54	0.590
	BF3	0.11	0.036	3.02	0.003 *			
	BF3 × Hit	-0.10	0.037	-2.76	0.006 *			
	BF3 × Fail	-0.03	0.040	-0.74	0.457	0.07	2.71	0.007 *
	BF4	-0.08	0.031	-2.39	0.017			
	BF4 × Hit	0.11	0.033	3.40	0.001 *			
	BF4 × Fail	0.12	0.035	3.47	0.001 *	0.01	0.50	0.617
	BF5	-0.05	0.024	-2.20	0.028			
	BF5 × Hit	0.10	0.025	3.98	< 0.001 *			
	BF5 × Fail	0.14	0.027	5.07	< 0.001 *	0.04	2.10	0.036
	BF6	0.00	0.024	-0.13	0.900			
	BF6 × Hit	0.04	0.025	1.50	0.132			
	BF6 × Fail	-0.01	0.026	-0.57	0.571	-0.05	-3.52	<0.004 *

* Significance based on FDR correction (<0.05), originalp-values are presented.

The largest peak differences were observed in a superior parietal subnetwork, the inferior frontal gyrus, and a paracingulate subnetwork. As presented in panel A ofFigure 8, the superior parietal subnetwork was suppressed more strongly during unsuccessful trials, whereas the inferior frontal subnetwork (panel B) and paracingulate subnetwork (panel C) were more strongly allocated. Refer toSupplementary Table S3for the statistical details of these models.

**Fig. 8.**
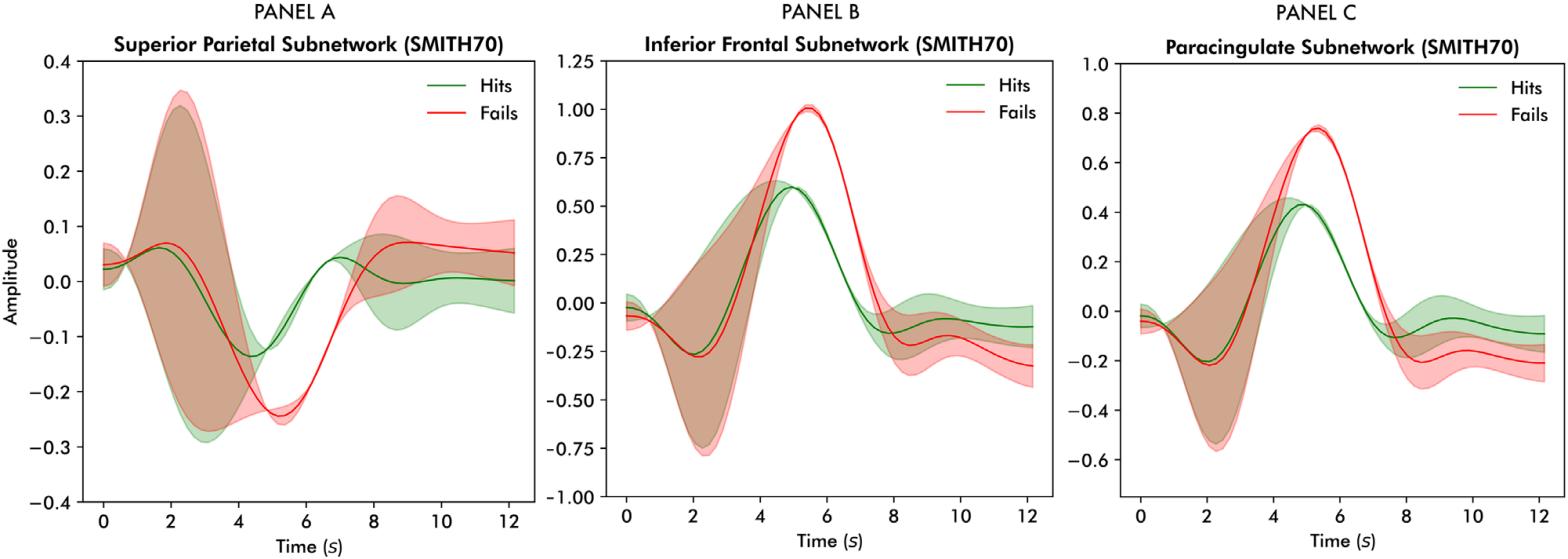
Reconstruction of allocation disparities between successful and unsuccessful trials based on the general linear models predicting subnetwork allocation from the FLOBS basis functions, task success, and their interactions for panel (A) superior parietal, panel (B) inferior frontal, and panel (C) paracingulate subnetworks.

## 4 Discussion

This paper presents TRIFLE, a time-resolved version of TFM analysis (Smith et al., 2012) that elucidates the moment-by-moment allocation of spatial sources associated with various TFMs. TRIFLE temporally unfolds the originally time-invariant linear mixing between spatial and temporal modes of organisation per time point, providing time-resolved, instantaneous functional loci estimates. We analytically demonstrated that instantaneous estimates of these functional loci reconfigurations can be retrieved in closed form via a time-resolved mixing matrix and that the single, fixed mixing matrix in classical TFM analysis corresponds to the temporal average of this time-varying quantity.

We provided an initial validation for the method by applying it to high temporal-resolution, task-based fMRI data. We concur withHuotari et al. (2019)that for time-resolved analyses, high-temporal resolution data (i.e., ideally with a TR<0.3 s) are preferable because it increases statistical power at the individual level; it provides more accurate signal distributions for statistics; it enables critical sampling and better characterisation of the physiological signals, and minimises aliasing of the physiological signal. While the sample size was relatively small, it did not hinder our objective of validating the TRIFLE model by demonstrating its ability to capture task-relevant network reconfigurations at the single-participant level, rather than generalising findings to the population. Applying the model at the single-participant level was made possible by the ultra-fast fMRI sequence used in this study, which provided the required temporal resolution. The importance of temporal resolution for the broader applicability of TRIFLE is revisited in a later section. First, we showed that the spatial source allocation for task-related TFMs is congruent with the expectations based on the task design. Specifically, across participants, task runs, and trials, we observed an increasing difference from baseline for the visual and subsequently for sensorimotor and auditory network weights. The temporal signatures of the latter two networks strongly overlapped, which is in line with the fact that the auditory feedback was given immediately after the motor response. In line with the well-supported role of the DMN as a task-negative network, we observed a relative deactivation of the precuneus subnetwork in response to the task.Singh and Fawcett (2008)previously showed that the strongest suppression of the DMN occurs for the most error-prone tasks. Congruently, in the second validation probing differences in functional loci allocation between successful and unsuccessful trials, we found stronger suppression of the precuneus subnetwork for unsuccessful trials compared with successful trials. As previously described, larger peak differences were observed in the superior parietal, inferior frontal gyrus, and paracingulate subnetworks. These are regions that have previously been implied in visuomotor association. The superior parietal lobe has been described as a critical integrator, binding visual, motor, and somatosensory information (Passarelli et al., 2021). Assuming that successful trials equate to better learned visuomotor associations, our finding that allocation of the inferior frontal gyrus is less strong for successful than for unsuccessful trials is congruent with the findings byToni et al. (2001)that this area shows a decrease in activity over time in response to learning.

In this study, we focused on single task-relevant TFMs. This was decided as we examined the congruency between network allocations and the task design for basic sensorimotor processes that rapidly succeeded each other. Doing so also solved the correspondence problem of temporal ICA. For more cognitively involved paradigms, we anticipate that different aspects of the task will be captured in different TFMs. We, therefore, suggest including several continuous cognitive, behavioural, or physiological regressors related to different processes of interest. Our decision to focus on task-relevant TFMs relates to an important assumption of TRIFLE, that is, that temporal ICA at stage two of the analysis adequately identifies the process of interest. Recall that with TRIFLE, instantaneous correlations between the individual spatial maps regressed onto the fMRI data and the TFM time series (scaled by their covariance) are retrieved. If a process of interest is not captured adequately using classical TFM analysis, the task-relevant information is unexplained residual variance. Using the same experimental paradigm,Gomez et al. (2020)previously found that temporal ICA captured the task design well. Conceptually, temporal ICA should indeed be well suited for capturing cognitive processes of interest, given that it optimises for temporal independence of sources. Temporal ICA is, however, not very common in fMRI as it requires many samples. Hence, the ability to adequately capture TFMs specific to certain task designs requires further validation.

We found support for the time-resolved mixing matrices capturing task-relevant information across participants. By averaging across trials, task runs, and participants, for this initial validation, we selectively focused on*within-trial*functional loci allocations and differences between successful and unsuccessful trials. Panel B ofFigures 6and7, as well as all ofFigure 8, illustrates that during the task, there were periods of synchronised network allocation across participants, interspersed with periods of significant interindividual variation in responses. In line with the shift that has occurred, moving from identifying group-level patterns of functional connectivity towards investigating individual differences (Bijsterbosch et al., 2021), these differences can be explored as a feature of interest in future work. While we found support for the time-resolved mixing matrices capturing task-relevant information across participants, interpretation of the group-level statistics may have been affected by the previously mentioned correspondence problem. Although task-relevant TFMs spatially strongly overlapped across task runs and participants (see Supplementary Material Section “Spatial Correspondence of Task-Relevant TFMs”), residual differences between components may have influenced group-level findings. In future studies aiming to identify generalisable effects, we suggest using a dual regression-like approach for the temporal ICA at stage two of the analysis to mitigate this issue. An additional remark regarding this validation study is that we solely regressed the visual task regressor against the time-resolved mixing matrix entries instead of also including the motor responses. This was decided because of strong multicollinearity between the regressors (due to the instruction to respond as soon as possible) and because we were not interested in contrasting these processes but in probing the functional loci allocations as retrieved with TRIFLE in response to the task in general. Another potential objection to this validation study is that the temporal differences in network allocation might result from differences in the latency of the HRF between regions. However, it cannot be the sole driving effect, as we identified different allocation profiles within the same networks for successful and unsuccessful trials. HRF variability not driven by neuronal effects but stemming from local physiological differences (e.g., regional differences in vascular architecture) would not be expected to depend on condition type or trial outcome.

Whereas so far, we have addressed potential limitations of the validation using task-based fMRI, an important consideration for the TRIFLE method overall concerns the number of time frames required to adequately capture the functional loci allocations by TFMs of interest. The field of fMRI has been dominated by the use of spatial ICA over temporal ICA, as the former performs more robustly. This advantage stems partly from the generally higher number of samples in space (i.e., voxels) than in time (time frames).Smith et al. (2012), therefore, ran the temporal ICA at the group level, that is, on temporally concatenated data from 5 subjects, yielding 24,000 time points. Using an ultra-fast MESH-EPI fMRI sequence (TR = 158 ms),Gomez et al. (2020)ran TFM analysis at the individual level. The present study used a sequence with a TR of 0.206 s, providing the temporal resolution needed to apply TRIFLE at the single-task run level, that is, 3000 time frames per task run and 9000 per participant. Although methods for rapid sampling of fMRI data are increasingly available, this is not yet common in most studies. We specifically probed the temporal limits of TRIFLE by downsampling the data and subsequently examining the component variances and group-level results. Based on the finding byHerrmann and Theis (2007)that the recovery error of the FastICA algorithm decreases with the inverse of the square root of the number of samples,Gomez et al. (2020)argued for the quite substantial minimal number of 3000 samples. Examining the component variances and group-level network allocation for different downsampling rates (presented in Supplementary Material Section “Temporal Limits of TRIFLE”) suggested that TRIFLE is applicable for temporal sample sizes on the scale of hundreds instead of thousands. This discrepancy warrants further exploration, preferably using simulation studies such that the true sources are known and reconstruction errors can be calculated.

Another key consideration for TRIFLE concerns its ability to differentiate between two distinct spatiotemporal phenomena observed in the very low-frequency (VLF) range of BOLD signals: standing waves and moving waves. Standing waves represent stationary oscillations with zero-lag synchrony, while moving waves involve propagative dynamics characterised by time-lagged synchrony (Kiviniemi et al., 2016;Q. Wang et al., 2021). The first step of TRIFLE, that is, spatial ICA, assumes stationarity in the spatial domain, making it well suited for capturing standing waves and static spatial distributions of vasomotor fluctuations (Kiviniemi et al., 2003) but less suited for identifying moving waves. However, moving waves may still leave residual traces in the spatial ICA time series if their dynamics are spatially structured and persist throughout the scanning duration. If sufficiently strong, these traces can be captured by temporal ICA, which jointly analyses all spatial ICA time series and does not impose a spatial stationarity constraint. As a result, TRIFLE’s time-varying mixing matrix can reflect both phenomena, although its reliance on spatial ICA introduces a bottleneck. However, spatial ICA is not a prerequisite of the model. In principle, TRIFLE is compatible with different spatial parcellations, ranging from other multivariate decompositions to time series extracted from standard anatomical parcellations or voxel time series for a region of interest. Future research could explore such alternative workflows.

The overarching aim of fMRI is to summarise large amounts of data that, among other factors, reflect changes in (T2*) relaxation due to changes in brain metabolism in a way that provides useful (i.e., predictive and biologically congruent) information on cognition and behaviour. We can evaluate time-varying functional connectivity methodologies by how they deal with this dimensionality reduction problem. As previously described, reductions in the temporal domain have been performed using larger time windows or removing temporal ordering at certain stages of the analysis. Building upon the strengths of TFM analysis, TRIFLE estimates temporal components by considering the entire network time series simultaneously rather than relying on windowed or instantaneous measures. This approach ensures that the full range of frequency variations in the temporal profiles is preserved. Traditional TFM analysis addresses the presence of noise in the linear mixing of spatial and temporal components through implicit averaging. In contrast, TRIFLE explicitly formalises this process, allowing for selective post hoc averaging across scans or time windows. This ensures that transitions in the linear mixing are not obscured a priori but can instead be identified and analysed. The findings byBijsterbosch et al. (2019)andBijsterbosch et al. (2018)that spatial overlap can bias functional connectivity estimates emphasise the influence that reductions in the spatial domain (i.e., parcellations) that precede the TVFC estimation or modelling can have on the validity of functional connectivity estimates. Using crude anatomical parcellations like the AAL, for example, TVFC metrics are computed from signals that likely consist of contributions from several functional connectivity processes, potentially obscuring the true underlying functional organisation. Of note, spatial overlap is not just an issue for functional connectivity-based analyses but also for mass-univariate GLM-based analyses, as unknown mixtures of network contributions to the voxel time series cannot be separated and, therefore, go unnoticed (Xu, Potenza, & Calhoun, 2013;Xu et al., 2016). We believe that TRIFLE provides relevant novel information while increasing biological validity compared with existing methodologies. Because fMRI data are intrinsically noisy in both temporal and spatial domains, complicating the estimation of non-stationarities, TRIFLE does not explicitly model spatial or temporal non-stationarity. Instead, it assumes that multivariate dimensionality reductions in the spatial and temporal domains of TFM analysis provide reliable estimates of independent spatial and temporal processes. By subsequently temporally unfolding the originally time-invariant linear mixing between them per time point, TRIFLE introduces a novel feature: time-resolved instantaneous functional loci estimates. TRIFLE probes these interactions at the highest possible (i.e., instantaneous) temporal resolution while considering temporal ordering.

To conclude, we presented a novel multivariate functional connectivity method that captures dynamic network reconfigurations while accounting for spatial overlap and provided an initial validation. The method retrieves time-resolved instantaneous estimates of the extent to which spatial sources are reconfigured and allocated by specific processes of interest. We believe it has the potential to elucidate novel spatiotemporal properties of functional network allocation and their role in cognition, behaviour, psychopathology, and neurodivergence.

## Supplementary Material

Supplementary Material

## Data Availability

Data will be stored at the Donders Repository (https://data.donders.ru.nl/) and are made available upon request. The GitHub repository has been put online. Moreover, please change this sentence to: A documented Python package for the TRIFLE method is available athttps://github.com/tamarajedidja/trifle. The package only supports Python 3.6 and later.
