# Supplementary material for: Time-resolved instantaneous functional loci estimation (TRIFLE): Estimating time-varying allocation of spatially overlapping sources in functional magnetic resonance imaging
Source: Imaging Neurosci (Camb). 2025 Jun 27;3:IMAG.a.58. doi: 10.1162/IMAG.a.58 (PMC12319831; doi:10.1162/IMAG.a.58)
Supplement: Supplementary Material [file imag.a.58_supp.pdf]

Supplementary Material for Time-Resolved Instantaneous Functional Loci Estimation (TRIFLE):

Estimating Time-Varying Allocation of Spatially Overlapping Sources in Functional Magnetic Resonance Imaging

Tamara Jedidja de Kloe,[1,2a] Zahra Fazal,[1] Nils Kohn,[2] David Gordon Norris,[1,3]

Ravi Shankar Menon,[4] Alberto Llera,[1,2b] Christian Friedrich Beckmann[1,2,5ab]

[1]Donders Institute, Radboud University, Nijmegen, The Netherlands
[2]Department for Cognitive Neuroscience, Radboud University Medical Center Nijmegen, Nijmegen, the Netherlands
[3]Erwin L. Hahn Institute, University Duisburg-Essen, Essen, Germany
[4]Robarts Research Institute, London, Ontario, Canada
[5]Oxford Centre for Integrative Neuroimaging, FMRIB, University of Oxford, Oxford, United Kingdom
[a]Correspondence: tamara.dekloe@donders.ru.nl, c.beckmann@donders.ru.nl
[b]Shared last authorship

SUPPLEMENTARY MATERIAL

EPI Image

Figure S1 shows a slice of a pre-processed EPI image acquired with the ultra-fast MESH-EPI fMRI sequence (TR = 206ms) for illustration purposes.

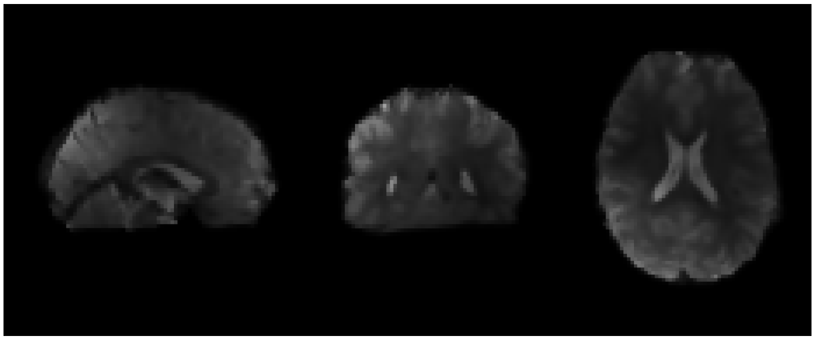

Figure S1: Single slice of a pre-processed EPI image obtained with the ultra-fast MESH-EPI fMRI sequence.

Association TFM Time series and Confounds

TFM time series that showed the strongest absolute correlation to the task regressors were correlated to confound regressor time series as an indication of their neuronal basis. The Fisher-Z transformed correlation values are shown for all task runs and participants in Figure S2. Evident from this figure is the enlarged associations with the global signal. This finding is in line with the conclusion by Glasser et al. (2018), that spatial ICA (as used for data cleaning in the current study) is very effective at removing spatially specific structured noise from high temporal resolution fMRI data but that it cannot selectively and completely remove global structured noise while retaining global signal from neural activity. Clean-up using temporal ICA was proposed as a potential solution.

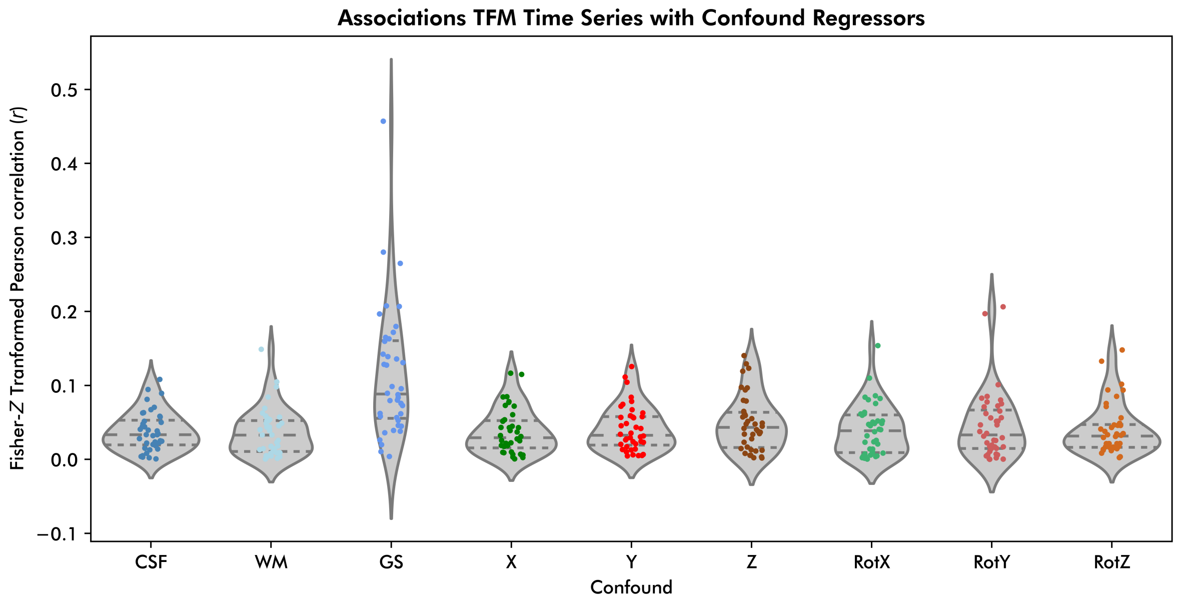

Figure S2: The distributions of the Fisher-Z transformed correlation values between the time series of the task-relevant TFM and the confound regressors per task run.

Identified TFMs

The most common TFM across task runs and participants was identified in a manner similar to Gomez et al. (2020), i.e., spatially correlating the time-invariant mixing matrix weights of all TFMs for the SMITH20 template and counting the number of correlation values surviving the threshold of $r = 0.49$, which was the smallest maximum correlation value per TFM across task runs. The most common TFM identified across task runs and participants resembled the *default temporal mode*, as identified by Gomez et al. (2020). It includes core hubs of the DMN (i.e. angular gyrus and precuneus) in anti-correlation with motor regions (superior parietal cortex, post-central gyrus and supplementary motor cortex), the auditory network, and the insula (see Figure S3).

Similar TFMs were identified across participants and task runs. The average spatial correlation of these TFMs with the default temporal mode for the selected dataset was $r = 0.61$ (SD $= 0.07$, range: 0.49–0.79). Panel A of Figure S4 shows two spatial maps of TFMs that correlated most strongly to the default temporal mode identified for the selected dataset. Contrary to the findings by Gomez et al. (2020), no anti-correlations with visual areas were found for some participants, including the selected dataset. This likely results from component splitting. For example, for the selected dataset, a temporal ICA of model-order ten did identify a component similar to the default temporal mode *also in anti-correlation to visual areas* (see panel B of Figure S4).

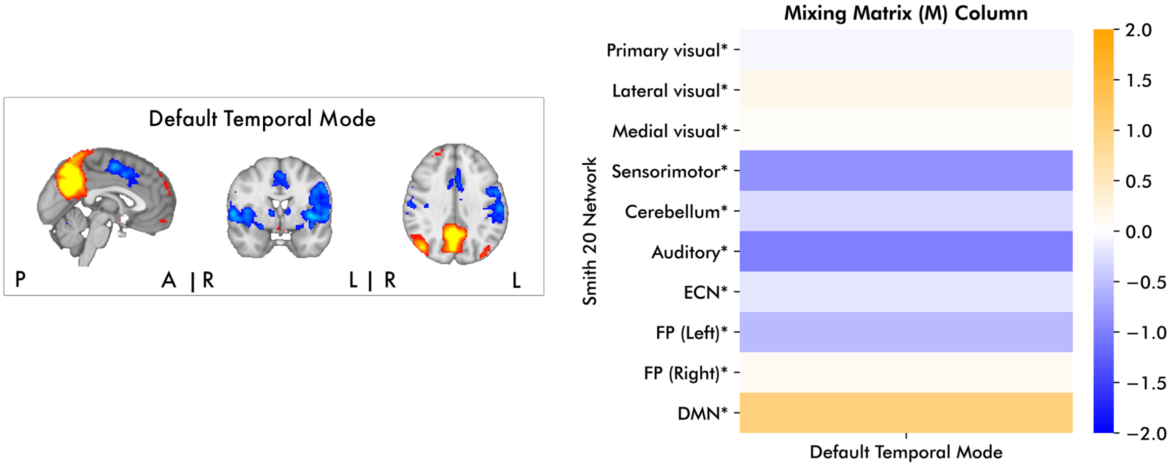

Figure S3: TFM spatial map: Three representative slices of the default temporal mode for the selected dataset (thresholded at Z 5) and the associated mixing matrix column representing the time-averaged weighting of this TFM onto the spatial sources (the ten well-defined SMITH20 networks). Abbreviations: P = posterior, A = anterior, L = left, R = right. ECN = executive control network, FP = frontoparietal network, DMN = default mode network.

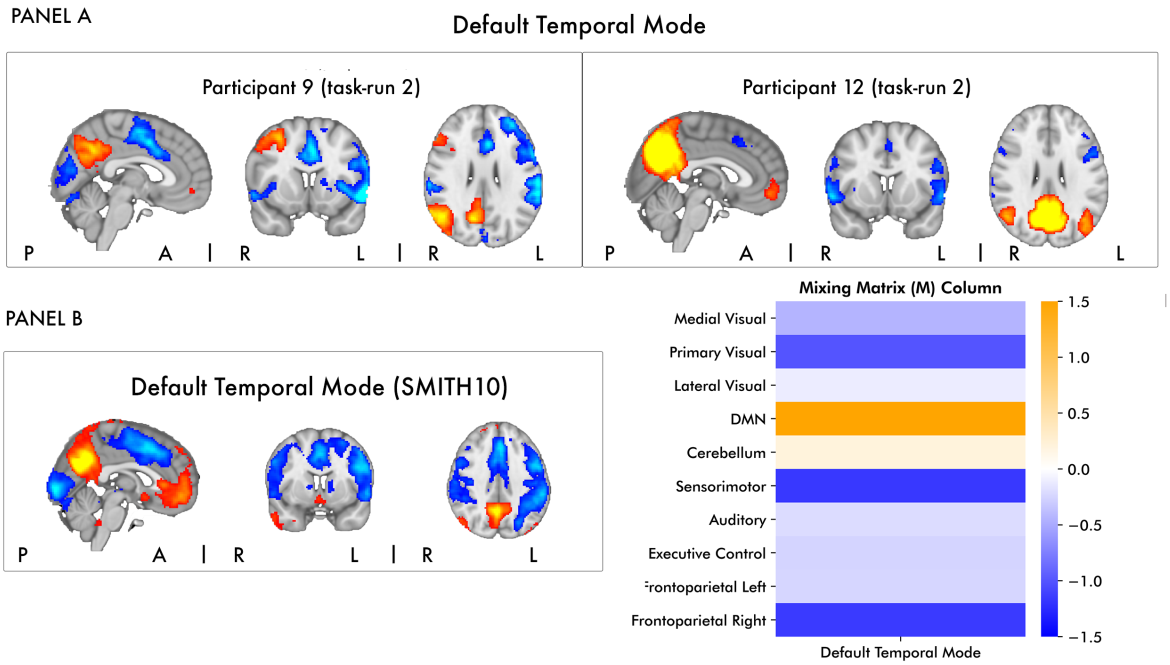

Figure S4: The two TFMs most strongly correlated to the default temporal mode as identified for the selected dataset are presented in panel A. Panel B shows three representative slices of the default temporal mode for the selected dataset after temporal ICA with a model order of ten (TFM1; thresholded at Z 5) and the associated mixing matrix column, which describes the time-averaged weighting of this TFM onto the spatial network template. Colour coding spatial maps: blue (negative) to red/yellow (positive).

Temporal Autocorrelation Lag

To determine the appropriate autoregressive lag for our Generalised Least Squares models with an autoregressive covariance structure, we computed the Partial Autocorrelation Function (PACF) for each network's time-varying mixing matrix time series using the statsmodels library in Python. The optimal lag length was identified by examining the PACF plots, focusing on where the PACF values exhibited a pronounced drop-off (see Figure S5). This resulted in the decision to use an autoregressive lag of 5.

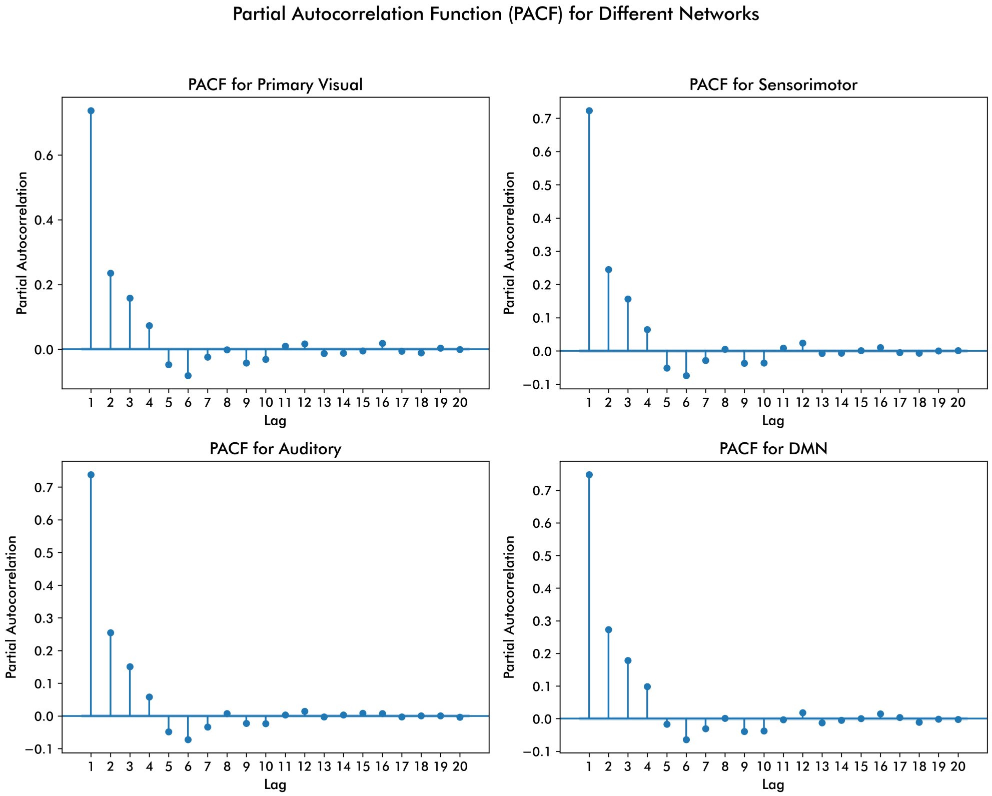

Figure S5: Partial Autocorrelation Functions (PACF) for the Primary Visual network, the Sensorimotor network, the Auditory network, and the Default Mode Network (DMN). Each plot displays the partial autocorrelation at different lags, showing the relationship between the current value and the value at a given lag while considering and accounting for the influence of all shorter lags.

Dataset Selection for Illustrative Purposes

Data from a single task run were selected for illustrative purposes. Given the model's assumption that temporal ICA at stage two of the analysis needs to identify the process of interest adequately, we selected the TFMs based on their temporal association with the visual task regressors. Among participants with all three runs, the selected dataset showed the strongest correlation ($r = 0.48$).

Trial-Based Statistics Network– and TFM Time Series

To illustrate how the spatial ICA output, the temporal ICA output and TRIFLE's time-resolved mixing of the two relate to one another, we trial-averaged the network– and TFM time series in the same way as we did for the time-varying mixing matrix for the selected dataset. Panel A of Figure S6 presents the trial-averaged network time series, and Panel B shows the trial-averaged TFM time series for the three most strongly involved TFMs. Panel C shows the time-invariant network involvement for the task-related TFM (i.e., TFM3) retrieved with TFM analysis. Panel D presents the network reconfigurations for the task-related TFM retrieved with TRIFLE. Panel D thus provides the temporally unfolded mapping of the time-invariant mapping as presented in panel C. Remember that: 1) the time-resolved mixing matrix concerns the element-wise product of the participant-specific expression of the network time series (i.e., AX) and the TFM time series (i.e., $B^{\dagger}$), scaled through Z, i.e., the covariance of B, and 2) that for normalised time series this amounts to the instantaneous correlation (van Oort et al., 2018).

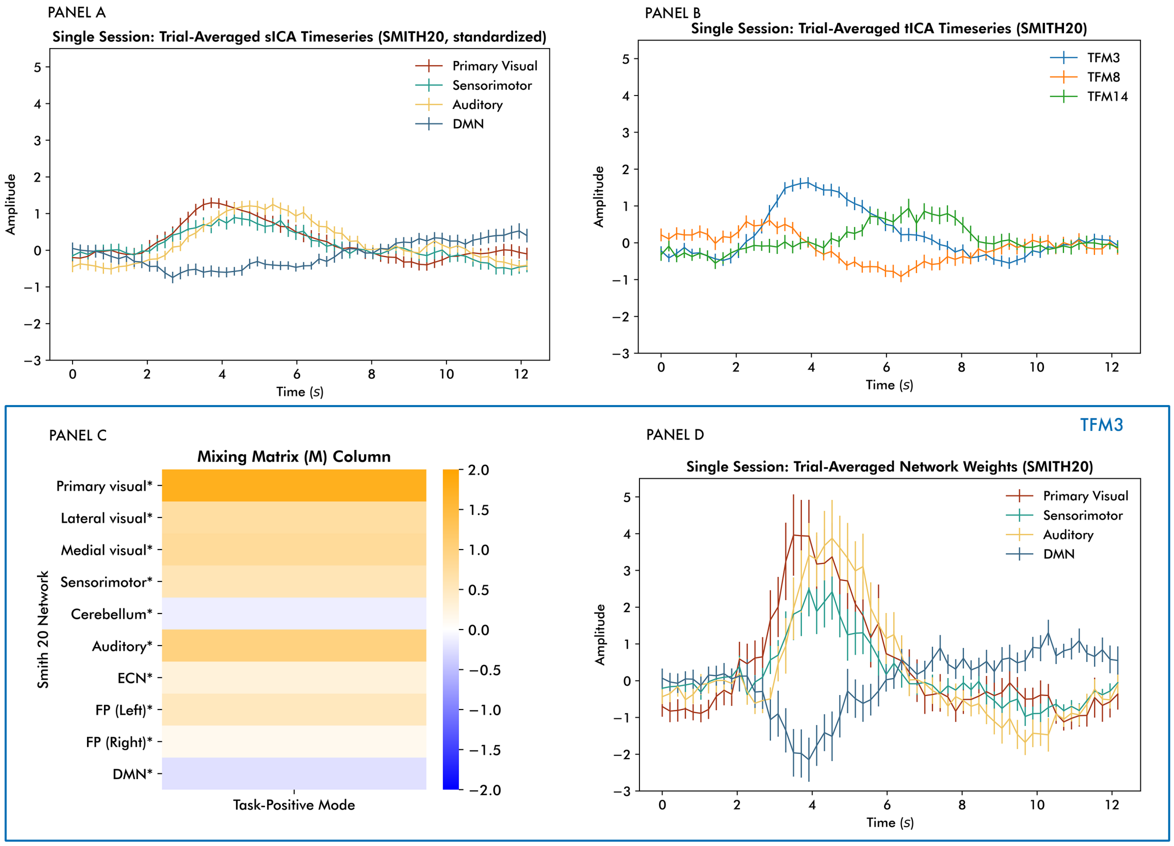

Figure S6: Trial-based statistics for: the network time series retrieved with spatial ICA (panel A), the TFM time series retrieved with temporal ICA (panel B), and the network reconfigurations for the task-relevant TFM (TFM3) retrieved with TRIFLE, for the selected dataset. Panel C shows the time-invariant mapping of the task-relevant TFM retrieved with TFM analysis, contrasting with the time-resolved mapping presented in panel D retrieved with TRIFLE.

Trial-Averaged Statistics From Motor Response

The trial-averaged statistics in the paper do not account for differences in response times. For comparison, we ran the same analysis with trials aligned to the motor response, starting each trial $10 \times TR$ before the response instead of the visual stimulus onset. This was chosen because the visual stimulus lasted 200ms, and participants had 1.5 seconds to respond. Results (see Figure S7) are similar to those presented in Figures 4 and 5 of the main manuscript, with slightly higher Z-values for the sensorimotor network, as expected due to the alignment with the motor response.

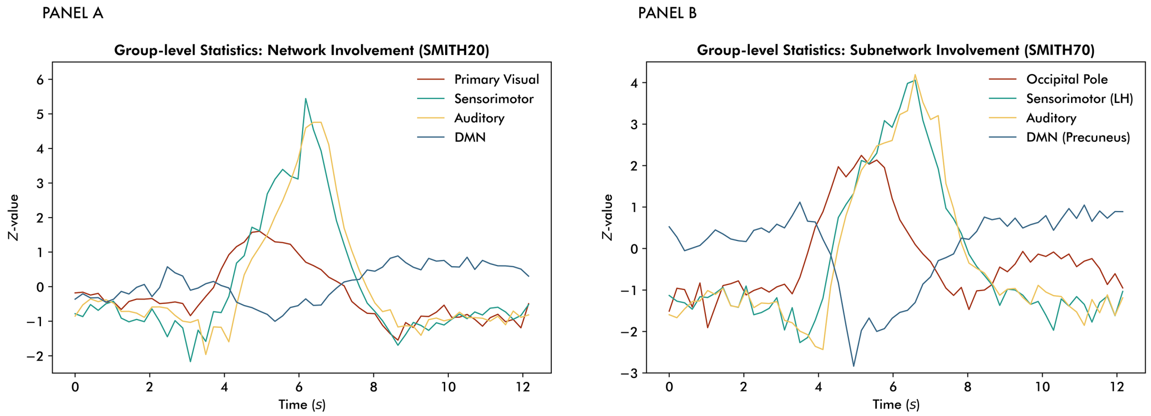

Figure S7: Group-level Z-distributions of differences from baseline for the primary visual, sensorimotor, auditory and default mode network where the onset is defined as 10*TR before the motor response for the functional networks (Panel A) and –subnetworks (Panel B).

GLM Statistics

Tables S1 and S2 show the GLM statistics predicting network and subnetwork allocation from the six FLOB basis functions. Original p-values are reported, with significance determined by FDR correction at a 0.05 threshold (indicated by *).

Table S1: GLM Results Predicting Network Allocation from FLOB Basis Functions.

**GLM Statistics**					
Outcome	Predictor	*b*	*SE*	*t*	*p*
Primary	BF1	0.08	0.017	4.53	<0.0001*
Visual	BF2	0.14	0.013	10.45	<0.0001*
	BF3	-0.02	0.013	-1.59	0.113
	BF4	-0.05	0.012	-4.59	<0.0001*

Continued on next page

Table S1 – *Continued from previous page*

GLM Statistics					
Outcome	Predictor	*b*	*SE*	*t*	*p*
	BF5	-0.07	0.011	-6.43	<0.0001*
	BF6	-0.04	0.009	-4.81	<0.0001*
	F-value: 70.27	p-value: <0.0001*			
Sensorimotor	BF1	0.18	0.018	9.54	<0.0001*
	BF2	0.14	0.014	9.42	<0.0001*
	BF3	-0.09	0.014	-6.49	<0.0001*
	BF4	-0.11	0.013	-8.49	<0.0001*
	BF5	-0.10	0.012	-8.28	<0.0001*
	BF6	0.00	0.010	0.351	0.726
	F-value: 124.80	p-value: <0.0001*			
Auditory	BF1	0.27	0.024	11.02	<0.0001*
	BF2	0.16	0.019	8.33	<0.0001*
	BF3	-0.18	0.019	-9.63	<0.0001*
	BF4	-0.15	0.017	-9.09	<0.0001*
	BF5	-0.16	0.016	-10.40	<0.0001*
	BF6	0.08	0.013	6.00	<0.0001*
	F-value: 174.10	p-value: <0.0001*			
DMN	BF1	-0.02	0.020	-0.97	0.334
	BF2	-0.06	0.016	-3.86	<0.0001*
	BF3	0.03	0.015	2.12	0.034*
	BF4	0.01	0.014	0.48	0.631
	BF5	0.03	0.012	2.19	0.028*
	BF6	0.00	0.010	0.41	0.686
	F-value: 6.92	p-value: <0.0001*			

Table S2: Results of the GLMs Predicting Subnetwork Allocation from the FLOBS basis functions .

GLM Statistics					
Outcome	Predictor	*b*	*SE*	*t*	*p*
Occipital	BF1	0.08	0.008	10.32	<0.0001*
Pole	BF2	0.06	0.006	10.21	<0.0001*
	BF3	0.03	0.007	4.08	<0.0001*
	BF4	-0.03	0.006	-4.85	<0.0001*
	BF5	-0.04	0.005	-7.44	<0.0001*

Continued on next page

Table S2 – *Continued from previous page*

GLM Statistics					
Outcome	Predictor	*b*	*SE*	*t*	*p*
	BF6	-0.05	0.004	-12.51	<0.0001*
	F-value: 128.20	p-value: <0.0001*			
Sensorimotor (RH)	BF1	0.04	0.009	4.81	<0.0001*
	BF2	0.02	0.007	2.67	0.008*
	BF3	0.00	0.008	-0.36	0.722
	BF4	-0.03	0.008	-3.50	<0.0001*
	BF5	-0.02	0.006	-2.88	0.004*
	BF6	-0.01	0.005	-2.43	0.015*
	F-value: 20.45	p-value: <0.0001*			
Sensorimotor (LH)	BF1	0.24	0.013	19.39	<0.0001*
	BF2	0.04	0.010	3.87	<0.0001*
	BF3	-0.07	0.011	-6.90	<0.0001*
	BF4	-0.09	0.011	-8.82	<0.0001*
	BF5	-0.10	0.008	-11.77	<0.0001*
	BF6	-0.01	0.006	-2.23	0.026*
	F-value: 254.50	p-value: <0.0001*			
Auditory	BF1	0.33	0.013	24.66	<0.0001*
	BF2	0.04	0.011	3.68	<0.0001*
	BF3	-0.10	0.012	-8.56	<0.0001*
	BF4	-0.15	0.012	-12.73	<0.0001*
	BF5	-0.13	0.009	-14.36	<0.0001*
	BF6	0.00	0.007	-0.48	0.632
	F-value: 378.90	p-value: <0.0001*			
Precuneus (DMN)	BF1	-0.12	0.015	-7.86	<0.0001*
	BF2	-0.03	0.011	-2.86	0.004*
	BF3	0.04	0.013	3.09	0.002*
	BF4	0.03	0.012	2.47	0.013*
	BF5	0.06	0.009	6.00	<0.0001*
	BF6	0.02	0.007	2.43	0.015*
	F-value: 48.23	p-value: <0.0001*			

Trial-Averaged Entries of f per Task Run, per Participant.

Figures S8 and S9 show the trial-averaged network and subnetwork allocations for the task-relevant TFM per task run and participant, respectively.

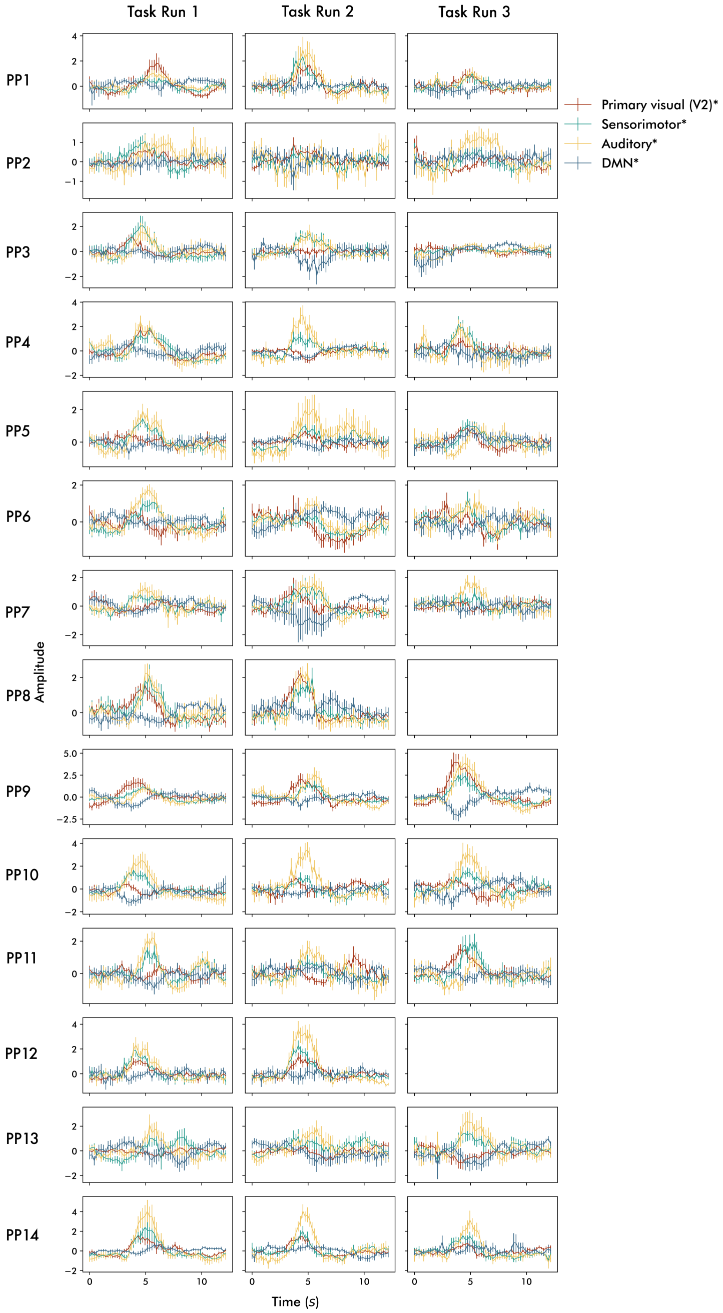

Figure S8: Trial-averaged entries of f for the task-relevant networks for the TFM that showed the strongest absolute correlation with the visual task regressor per task run and participant.

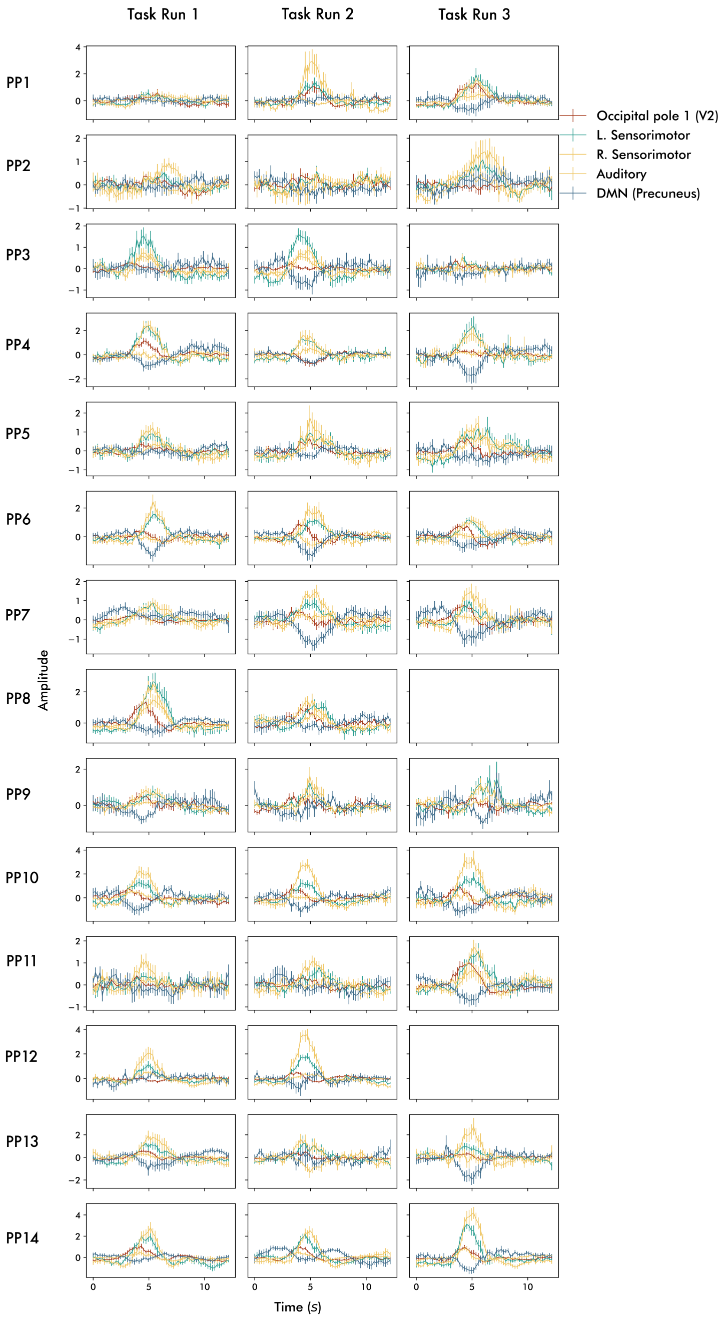

Figure S9: Trial-averaged entries of f for the task-relevant subnetworks for the TFM that showed the strongest absolute correlation with the visual task regressor per task run and participant.

Statistics General Linear Models Regarding Task Success.

Table S3 presents the statistics of the general linear models predicting subnetwork allocation from the six FLOBS' basis functions, task success, and the interactions with the six FLOBS' basis functions for the three subnetworks, which showed the largest peak differences between successful and unsuccessful trials using the trial-averaging approach.

Table S3: GLM Statistics from Models Predicting Network Allocation from FLOBS' basis functions and Task Success.

GLM Statistics						Post-hoc Contrasts Fail–Hit		
Outcome	Predictor	*b*	*SE*	*t*	*p*	Δ *b*	*t*	*p*
Superior	Hit	0.01	0.010	0.50	0.618			
Parietal	Fail	-0.02	0.014	-1.27	0.203	-0.02	-1.39	0.164
	BF1	-0.07	0.021	-3.43	0.001*			
	BF1 x Hit	0.04	0.022	1.85	0.065			
	BF1 x Fail	-0.01	0.024	-0.37	0.710	-0.05	-2.99	0.002*
	BF2	-0.14	0.029	-4.93	<0.0001*			
	BF2 x Hit	0.12	0.029	4.19	<0.0001*			
	BF2 x Fail	0.14	0.030	4.50	<0.0001*	0.01	1.04	0.298
	BF3	0.01	0.019	0.55	0.580			
	BF3 x Hit	-0.02	0.021	-0.92	0.358			
	BF3 x Fail	0.01	0.022	0.50	0.618	0.03	2.00	0.045
	BF4	-0.03	0.018	-1.65	0.098			
	BF4 x Hit	0.05	0.019	2.65	0.008*			
	BF4 x Fail	0.05	0.020	2.61	0.009*	0.00	0.22	0.822
	BF5	-0.03	0.014	-2.25	0.024			
	BF5 x Hit.	0.04	0.015	3.01	0.003*			
	BF5 x Fail.	0.04	0.016	2.75	0.006*	0.00	-0.04	0.969
	BF6	-0.04	0.014	-2.58	0.010*			
	BF6 x Hit	0.05	0.015	3.60	<0.0001*			
	BF6 x Fail	0.03	0.016	2.19	0.029	-0.02	-2.10	0.035
F-value: 10.04, p-value: <0.0001*								
Inferior	Hit	0.00	0.021	0.00	0.996			
Frontal	Fail	0.09	0.028	3.02	0.002*	0.09	2.66	0.008*
	BF1	0.24	0.040	5.84	<0.0001*			
	BF1 x Hit	-0.09	0.041	-2.27	0.024			
	BF1 x Fail	0.05	0.045	1.22	0.222	0.15	4.77	<0.0001*

Continued on next page

Table S3 – *Continued from previous page*

**GLM Statistics**						**Post-hoc Contrasts Fail–Hit**		
Outcome	Predictor	*b*	*SE*	*t*	*p*	Δ *b*	*t*	*p*
	BF2	0.03	0.054	0.65	0.516			
	BF2 x Hit	0.00	0.055	0.03	0.997			
	BF2 x Fail	-0.06	0.057	-1.03	0.304	-0.06	-2.47	0.013*
	BF3	-0.16	0.036	-4.58	<0.0001*			
	BF3 x Hit	0.10	0.037	2.70	0.007*			
	BF3 x Fail	0.05	0.040	1.27	0.206	-0.05	-1.85	0.065
	BF4	-0.09	0.032	-2.77	0.006*			
	BF4 x Hit	0.03	0.033	0.81	0.421			
	BF4 x Fail	0.04	0.035	1.11	0.268	0.01	0.54	0.587
	BF5	-0.07	0.024	-2.75	0.006*			
	BF5 x Hit	-0.02	0.025	-0.65	0.518			
	BF5 x Fail	-0.02	0.027	-0.85	0.397	-0.01	-0.38	0.706
	BF6	0.05	0.024	2.23	0.026			
	BF6 x Hit	-0.06	0.025	-2.57	0.010*			
	BF6 x Fail	0.01	0.026	0.22	0.830	0.07	4.69	<0.0001*
F-value: 44.18, p-value: <0.0001*								
Para–cingulate	Hit	0.00	0.014	-0.13	0.900			
	Fail	0.06	0.019	3.27	0.001*	0.06	2.92	0.003*
	BF1	0.17	0.027	6.18	<0.0001*			
	BF1 x Hit	-0.07	0.028	-2.51	0.012*			
	BF1 x Fail	0.04	0.031	1.25	0.212	0.11	5.15	<0.0001*
	BF2	0.07	0.037	1.82	0.069			
	BF2 x Hit	-0.04	0.038	-1.09	0.275			
	BF2 x Fail	-0.06	0.039	-1.46	0.143	-0.02	-0.95	0.340
	BF3	-0.08	0.025	-3.35	0.001*			
	BF3 x Hit	0.04	0.026	1.74	0.083			
	BF3 x Fail	-0.01	0.028	-0.38	0.706	-0.05	-2.97	0.003*
	BF4	-0.04	0.022	-2.00	0.045			
	BF4 x Hit	0.00	0.023	-0.19	0.849			
	BF4 x Fail	0.00	0.025	-0.15	0.884	0.00	0.04	0.964
	BF5	-0.04	0.017	-2.35	0.019*			
	BF5 x Hit	-0.03	0.017	-1.54	0.123			
	BF5 x Fail	-0.03	0.019	-1.59	0.112	0.00	-0.24	0.807

Continued on next page

Table S3 – *Continued from previous page*

GLM Statistics						Post-hoc Contrasts Fail–Hit		
Outcome	Predictor	*b*	*SE*	*t*	*p*	Δ *b*	*t*	*p*
	BF6	0.02	0.016	0.92	0.359			
	BF6 x Hit	-0.03	0.017	-1.52	0.128			
	BF6 x Fail	0.01	0.018	0.77	0.439	0.04	3.90	<0.0001*
F-value: 51.06, p-value: <0.0001*								

*) Significance based on FDR correction (< 0.05), original p-values are presented.

Spatial Correspondence of Task-Relevant TFMs.

Task-relevant TFMs were selected based on their temporal correlation to the task. Figure S10 presents heatmaps of the spatial correlation matrices between these TFMs per task run. Strong spatial overlap between most of the task-relevant TFMs was observed.

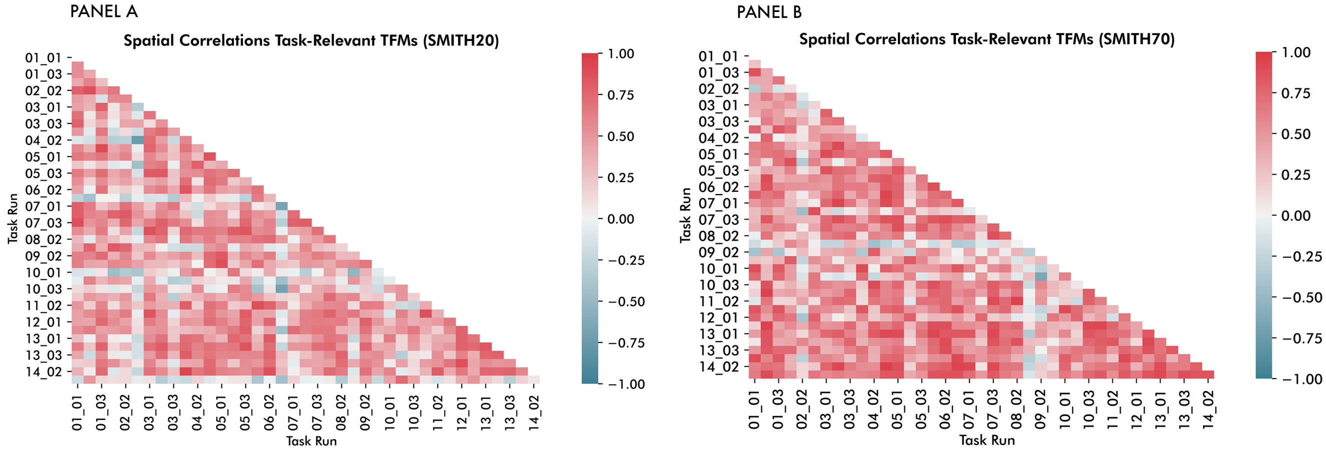

Figure S10: Spatial correlation heatmaps of the task-relevant TFMs for the network template (panel A) and subnetwork template (panel B), respectively.

Temporal Limits of TRIFLE.

Herrmann and Theis (2007) found that with an increase in the number of samples, the recovery error of the FastICA algorithm decreases in proportion to the inverse square root of the sample size. Based on the recommended number of frames required to achieve an average error of 0.1 as calculated by Herrmann and Theis (2007) across different levels of source kurtosis, Gomez et al. (2020) recommended a minimum of 3000 samples for TFM analysis. Herrmann and Theis (2007) used a different method for estimating negentropy than Gomez et al. (2020) and ourselves, however. While the former used a

Gram-Charlier expansion of densities, Gomez et al. (2020) and ourselves used the logcosh hyperbolic tangent. This approach may not rely as heavily on higher-order statistics.

Unlike in Herrmann and Theis (2007), recovery errors cannot be calculated for experimental data as the true underlying sources are unknown. Therefore, to probe the effects of decreased sample sizes, we examined 1) the component variances for increasing levels of downsampling and 2) network allocation as described by the time-resolved mixing matrix at the group level for increasing levels of downsampling.

Figure S11 shows the increase in component variances for increasing downsampling rates (i.e., smaller numbers of time frames). Due to a decreased signal-to-noise ratio, a larger variance is expected for reduced numbers of time frames. Notably, we observed that variances do not drastically increase for samples larger than ± 200 time frames. Moreover, we observed that the variances and their respective increases are consistent across components, resulting from the variance normalisation of the input data. As expected, this breaks down when the mixing matrix rank is degenerate, i.e., the number of time points is smaller than the model order of 15 in this case.

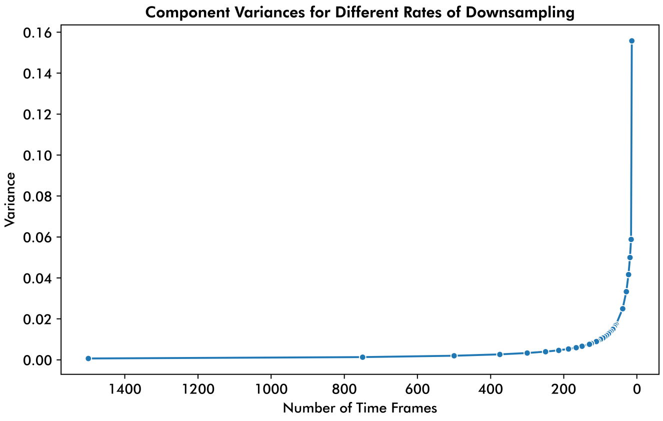

Figure S11: Component variances are presented for increasing levels of downsampling, i.e., increasingly smaller temporal sample sizes.

Figure S12 shows the group-level network allocation as described by the time-resolved mixing matrix for different downsampling rates. In addition to a general reduced power compared to our group-level results shown in Figure 4 of the main manuscript, the main effect is a reduction in temporal specificity for smaller numbers of time frames. Whereas the temporal pattern congruent with the task (i.e., visual stimuli presented for 200 ms, preparing and executing the motor response directly followed by auditory feedback) can still be disentangled reasonably well for $N = 750$ (panel B), this begins to break down at $N = 500$ (panel C). However, these sample sizes cannot be used as a general rule of thumb as they are contingent on this specific task and the associated effect sizes as well as the model order of the decompositions.

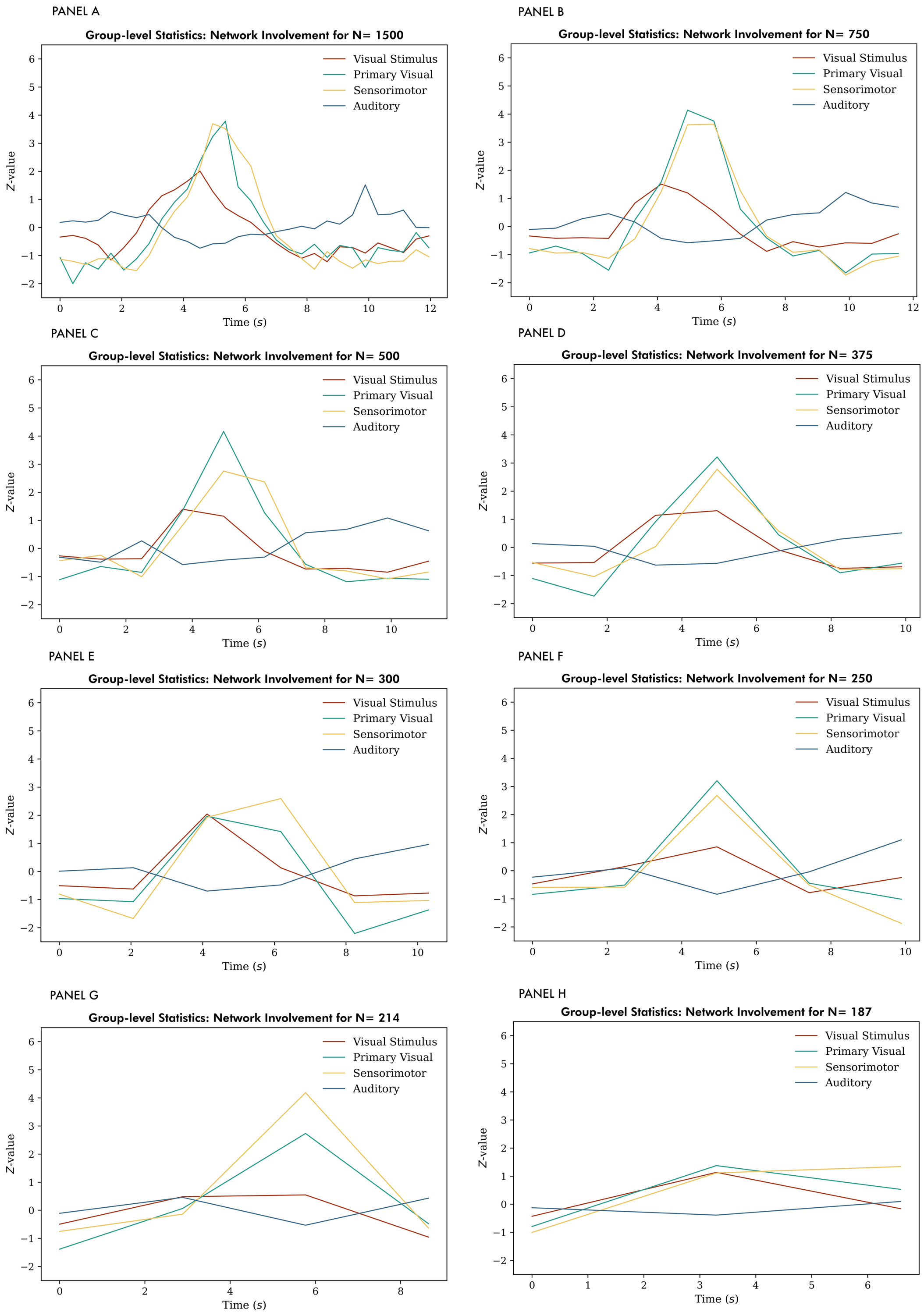

Figure S12: Each panel presents group-level differences from baseline for the primary visual, sensorimotor, auditory and default mode networks (indicated by the red, green, yellow and blue Z-value time series, respectively) for increasing downsampling rates.

These findings do, however, indicate that sample sizes smaller than suggested by Gomez et al. (2020) are eligible for the use of TRIFLE. This is congruent with the findings of Bielczyk et al. (2017), although this work specifically focused on effective connectivity. They examined under which conditions the effective connectivity problem becomes ill-posed. They found that relatively short time series (of a few hundred samples) suffice and that further upsampling does not improve the ability to retrieve the directionality of connections using lagged methods.

REFERENCES

Bielczyk, N. Z., Llera, A., Buitelaar, J. K., Glennon, J. C., & Beckmann, C. F. (2017). The impact of hemodynamic variability and signal mixing on the identifiability of effective connectivity structures in BOLD fMRI. *Brain and Behavior*, *7*(8), e00777. https://doi.org/10.1002/brb3.777

Glasser, M. F., Coalson, T. S., Bijsterbosch, J. D., Harrison, S. J., Harms, M. P., Anticevic, A., Van Essen, D. C., & Smith, S. M. (2018). Using temporal ICA to selectively remove global noise while preserving global signal in functional MRI data. *NeuroImage*, *181*, 692–717. https://doi.org/10.1016/j.neuroimage.2018.04.076

Gomez, D. E., Llera, A., Marques, J. P. F., Beckmann, C. F., & Norris, D. G. (2020). Single-subject, Single-session, Temporal Modes of Brain Activity. *NeuroImage*, 116783. https://doi.org/10.1016/j.neuroimage.2020.116783

Herrmann, J. M., & Theis, F. J. (2007). Statistical Analysis of Sample-Size Effects in ICA. In H. Yin, P. Tino, E. Corchado, W. Byrne, & X. Yao (Eds.), *Intelligent Data Engineering and Automated Learning - IDEAL 2007* (pp. 416–425). Berlin, Heidelberg, Springer Berlin Heidelberg. https://doi.org/10.1007/978-3-540-77226-2_43

van Oort, E. S., Mennes, M., Navarro Schröder, T., Kumar, V. J., Zaragoza Jimenez, N. I., Grodd, W., Doeller, C. F., & Beckmann, C. F. (2018). Functional parcellation using time courses of instantaneous connectivity. *NeuroImage*, *170*, 31–40. https://doi.org/10.1016/j.neuroimage.2017.07.027
